# Evaluation of early trabecular changes around implants using fractal analysis and panoramic indices

**DOI:** 10.1371/journal.pone.0352984

**Published:** 2026-07-01

**Authors:** Mert Keles, Ibrahim Burak Yuksel, Dilek Ozkan Sen, Omer Ogutcen, Muhammet Emin Arslan, Fatma Ucan Yarkac, Osman Babayigit

**Affiliations:** 1 Department of Periodontology, Faculty of Dentistry, Lokman Hekim University, Ankara, Turkey; 2 Department of Oral and Maxillofacial Radiology, Faculty of Dentistry, Necmettin Erbakan University, Konya, Turkey; 3 Department of Periodontology, Faculty of Dentistry, Necmettin Erbakan University, Konya, Turkey; Nigde University: Nigde Omer Halisdemir Universitesi, TÜRKIYE

## Abstract

Early trabecular changes around dental implants may not be adequately captured by conventional panoramic morphometric indices. Fractal analysis has been proposed as a quantitative method for detecting subtle changes in bone microarchitecture. This study aimed to evaluate early peri-implant trabecular structural changes using fractal analysis and panoramic morphometric indices during the unloaded healing period. This retrospective study included 60 single-tooth dental implants. Standardized panoramic radiographs were obtained at baseline and 3 months postoperatively. Mandibular cortical width, panoramic mandibular index, and fractal dimension values were measured in mesial, distal, and apical regions using ImageJ software. As all implants healed submerged and unloaded, periodontal parameters—plaque index, gingival index, bleeding on probing, and probing pocket depth—were recorded from natural teeth in the same quadrant. Baseline and 3-month values were compared using paired t-tests, with statistical significance set at p < 0.05. Fractal dimension values increased significantly in all evaluated regions at 3 months compared with baseline (p < 0.05), indicating measurable early trabecular structural changes. In contrast, mandibular cortical width and panoramic mandibular index showed no significant changes. Among periodontal parameters, only gingival index demonstrated a significant improvement (p < 0.001), reflecting reduced gingival inflammation. Fractal analysis detected early trabecular changes during the 3-month unloaded healing period, whereas conventional panoramic morphometric indices showed no significant change. These findings should be interpreted as indicators of trabecular reorganization rather than direct evidence of osseointegration. Fractal analysis may provide supplementary, non-invasive information during early radiographic follow-up.

## Introduction

Dental implant treatments are widely accepted for the rehabilitation of partially or completely edentulous jaws [1]. The long-term success of dental implants depends on several interrelated factors, including the patient’s systemic health, surgical approach, implant design, and, most importantly, the quality and quantity of the surrounding alveolar bone [2]. Among these factors, bone quality and primary implant stability are particularly important because they directly influence osseointegration, the biological process that enables a stable connection between the implant and the surrounding bone tissue [3].

To mitigate the risk of implant failure, preoperative assessment of bone quality is essential. Although methodologies such as dual-energy X-ray absorptiometry (DEXA), conventional computed tomography (CT), and cone-beam computed tomography (CBCT) have been suggested for this purpose, their application in routine dental practice remains constrained due to limitations including cost, radiation exposure, and restricted accessibility [4,5].

As a practical alternative, panoramic radiographs are frequently utilized in daily clinical practice because they offer low radiation exposure, are cost-effective, and are widely available [1]. Morphometric indices obtained from panoramic images, such as the mandibular cortical index (MCI), mandibular cortical width (MCW), and panoramic mandibular index (PMI), have demonstrated correlations with systemic bone conditions like osteoporosis [6]. These indices are also useful for estimating local bone quality [7]. In recent years, fractal analysis (FA) has gained attention as a complementary method for evaluating bone microarchitecture in dental radiographs. Originally introduced by Mandelbrot, FA is a mathematical approach that quantifies the structural complexity of trabecular bone [8,9]. Previous studies have shown that FA correlates with bone mineral density and may be useful for evaluating trabecular bone structure on dental radiographs. However, its value in detecting early peri-implant trabecular changes on panoramic radiographs has not been sufficiently investigated [10,11].

Although most previous studies have focused on long-term peri-implant bone changes, only a limited number have investigated early alterations during the initial healing phase [11,12]. For example, recent studies have evaluated peri-implant bone architecture during the early stages of implant treatment using fractal analysis, supporting the relevance of radiographic assessment during the unloaded healing period [11]. The first 3 months after implant placement represent an early unloaded healing period during which peri-implant trabecular structural changes may occur before more apparent cortical or morphometric changes become evident. Evaluating this interval may therefore provide useful information on early trabecular changes around implants. Clinical periodontal parameters were also included as secondary exploratory variables to provide additional clinical context for the local oral environment during early healing.

The primary aim of this study was to evaluate early peri-implant trabecular structural changes by comparing fractal dimension (FD) values derived from panoramic radiographs obtained at baseline and at 3 months postoperatively. Secondary exploratory analyses assessed these findings in relation to panoramic morphometric indices and clinical periodontal parameters. The null hypothesis was that there would be no significant difference in FD values between baseline and 3 months. No formal hypothesis was established for the secondary exploratory analyses involving panoramic morphometric indices and clinical periodontal parameters.

## Materials and methods

### Study procedure

This retrospective study adhered to the Declaration of Helsinki and was approved by the Necmettin Erbakan University Faculty of Dentistry Non-Drug and Non-Medical Device Research Ethics Committee (Approval No. 2025/639; 31.07.2025). As the study was based solely on anonymized retrospective data, individual informed consent was not obtained. Patient records, including anamnesis and periodontal evaluations, were obtained from the institutional archive. For the purposes of this research, the institutional archive was accessed between 20/08/2025 and 20/11/2025, and the relevant anonymized clinical and radiographic data were retrieved during this period.

A total of 60 patients (24 males, 36 females; age range: 21–74 years) were consecutively screened from the institutional archive and selected according to the inclusion and exclusion criteria. Each had standardized digital panoramic radiographs available at two time points: before implant placement (baseline) and at 3 months postoperatively (follow-up). Only individuals with complete radiographic records and posterior single-tooth implant sites were included in the study. At 3 months, healing abutments were placed, followed by definitive prosthetic restorations using custom abutments.

Demographic data, medical and dental histories, and periodontal clinical parameters—plaque index (PI), gingival index (GI), bleeding on probing (BOP), and probing pocket depth (PPD)—were obtained from routine clinical examination records. Since all implants healed submerged and unloaded during the first 3 months, these periodontal measurements were obtained from natural teeth in the same quadrant, not from the implants. These variables were evaluated as secondary exploratory clinical parameters and were used to provide clinical context for the radiographic findings rather than as direct measures of peri-implant healing. PI and GI were evaluated using the Silness-Löe and Löe-Silness criteria, respectively. PPD was measured at six sites per tooth using the UNC-15 periodontal probe (Hu-Friedy, Chicago, IL, USA) [13]. Radiographic gingivitis or periodontitis diagnoses were determined according to the 2017 World Workshop classification system [14]. For subgroup analyses, implants were categorized according to jaw location (maxilla/mandible) and implant length (8 mm/10 mm). Baseline periodontal status was classified as gingivitis or periodontitis according to the available clinical and radiographic records.

All implants were placed by the same experienced surgeon using a BIL® Dental Implant System (BIL Implant, Istanbul, Turkey) with diameters of 3.5 or 4.1 mm and lengths of 8 or 10 mm, without any bone grafting. Implants were placed following manufacturer guidelines.

Exclusion criteria included:

(1) Systemic diseases or medications affecting bone metabolism (e.g., osteoporosis, corticosteroids, bisphosphonates);
(2) Immediate implant placement or loading;
(3) Need for augmentation procedures;
(4) Tissue-level or partially exposed implant necks;
(5) Presence of multiple tooth loss or adjacent implants in the same region;
(6) Interfering structures (adjacent teeth, other implants, artifacts) within ROI areas;
(7) Poor-quality or distorted radiographs;
(8) Follow-up radiographs in which crestal bone loss, peri-implant radiolucency, or cortical disruption prevented reproducible placement of the predefined trabecular ROIs.

Cases were not excluded solely because crestal bone loss was clinically present; exclusion was applied only when radiographic bone loss or related radiographic changes prevented reproducible placement of the predefined trabecular ROIs without including cortical defects, radiolucent areas, the implant surface, or anatomical superimposition.

All panoramic radiographs were obtained using the same device (Planmeca ProOne®, Helsinki, Finland) and were acquired by a single trained radiology technician to ensure standardization. Exposure parameters were fixed for all patients (68 kVp, 7 mA, 10 s). Image pre-processing was performed using Romexis® software with fixed brightness, contrast, and filter settings.

Images were exported from RAW format and standardized to 3840 × 1872 pixels (600 dpi) using PhotoScape X Pro software (MOOII Tech). All analyses were performed on a calibrated full-HD monitor (24-inch, 1920 × 1080 px, 120 Hz; [Dell UltraSharp U2415]) under standardized room lighting conditions.

Calibration was ensured through repeated measurements on ten non-study cases. Two independent examiners (MEA and IBY) each performed the measurements twice on 10 randomly selected cases with a one-week interval, and both intra- and inter-observer reliability were assessed (ICC > 0.90). Agreement exceeded 95%.

### Morphometric measurements

**Fig 1:**
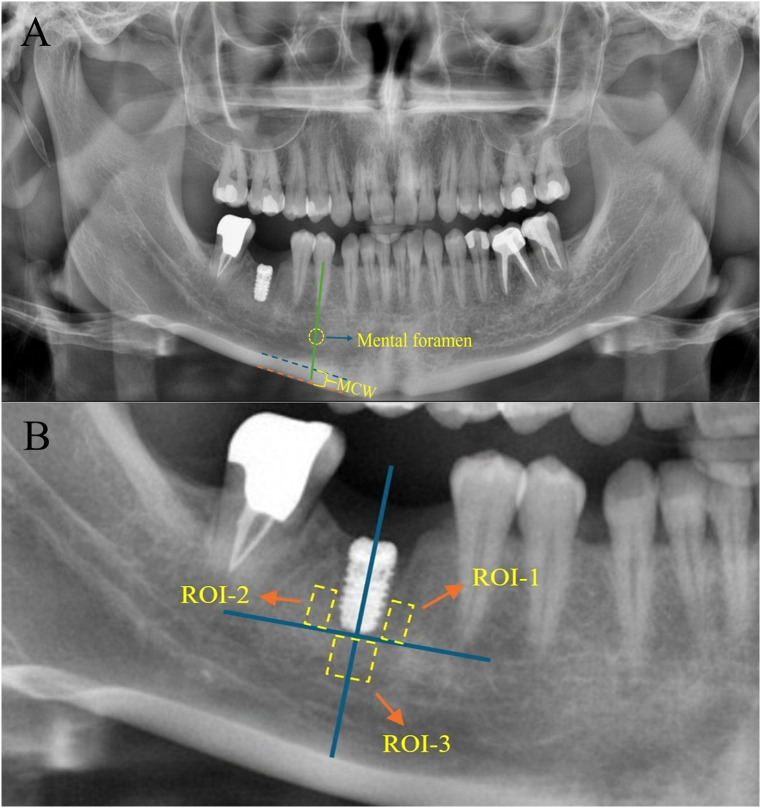
Standardized morphometric measurements and peri-implant ROI placement on panoramic radiography. (A) Morphometric measurements on panoramic radiography. The mental foramen was used as the anatomical reference point for mandibular cortical width (MCW) and mental length (ML) measurements. MCW was measured perpendicular to the inferior mandibular cortex, and PMI was calculated as the ratio of MCW to ML. (B) Enlarged view of peri-implant ROI placement. For fractal analysis, the blue longitudinal line indicates the implant long axis, and the blue transverse line indicates the perpendicular apical reference line passing through the implant apex. These geometric reference lines were defined on the 3-month radiograph and were used to reproduce the corresponding baseline ROI locations by side-by-side matching of stable anatomical landmarks. ROI-1 and ROI-2 were positioned in the closest available mesial and distal peri-implant trabecular bone adjacent to the implant surface, respectively, with their inferior borders aligned with the apical reference line. ROI-3 was positioned immediately apical to the implant apex, with its superior border aligned with the same apical reference line and centered on the implant long axis. The implant surface, implant threads, adjacent roots, cortical bone, mandibular canal, sinus floor, radiopaque restorations, and areas of anatomical superimposition or artifact were excluded from the ROIs.

Before FA, morphometric measurements, including mandibular cortical width (MCW), mental length (ML), and panoramic mandibular index (PMI), were performed on panoramic radiographs. MCW was measured perpendicular to the mandibular lower border at the midpoint of the mental foramen. ML was defined as the vertical distance from the lower edge of the mental foramen to the inferior mandibular cortex. PMI was calculated as the ratio of MCW to ML (Fig 1A). Because mandibular cortical and mental foramen-related measurements may show side-related variation on panoramic radiographs, MCW, ML, and PMI were evaluated separately on the right and left sides. Measurements were performed on TIFF images using ImageJ software (v1.54, NIH, Bethesda, MD, USA). Pixel-to-millimeter calibration was applied according to device specifications to minimize magnification errors. All morphometric measurements were independently performed by two examiners under standardized radiographic conditions.

### Fractal analysis procedure

Fractal analysis (FA) was performed using ImageJ software. For each implant, three peri-implant trabecular regions of interest (ROIs) were selected on baseline and 3-month panoramic radiographs: ROI-1 in the mesial peri-implant trabecular bone, ROI-2 in the distal peri-implant trabecular bone, and ROI-3 in the apical trabecular bone. ROI-1 and ROI-2 were fixed rectangular areas of 25 × 50 pixels, whereas ROI-3 was a fixed square area of 50 × 50 pixels. The same ROI dimensions were used for all implants and at both time points.

To improve reproducibility, baseline and 3-month panoramic radiographs were evaluated side by side. Because baseline radiographs were obtained before implant placement, the implant long axis and the perpendicular apical reference line passing through the implant apex were first defined on the 3-month radiograph using the visible implant contour and apex. ROI-1, ROI-2, and ROI-3 were then positioned on the follow-up radiograph according to the predefined ROI placement protocol. The corresponding baseline ROI locations were reproduced on the preoperative radiograph by matching the implant recipient site with stable anatomical and radiographic landmarks, including adjacent root contours, alveolar crest morphology, edentulous ridge configuration, surrounding trabecular pattern, and region-specific anatomical boundaries such as the maxillary sinus floor, mandibular canal, or mental foramen when present. Side-by-side assessment was used only to identify corresponding anatomical locations for ROI placement and not to visually judge changes in FD values.

ROI placement was standardized using the implant long axis as the primary orientation reference and the perpendicular apical reference line as the corono-apical reference. ROI-1 and ROI-2 were oriented parallel to the implant long axis, with their inferior borders aligned with the apical reference line. These ROIs were positioned in the closest available mesial and distal peri-implant trabecular bone adjacent to the implant surface, without including the implant threads or implant surface. ROI-3 was placed immediately apical to the implant apex, with its superior border aligned with the same apical reference line and centered on the implant long axis (Fig 1B).

During ROI placement, the implant surface, implant threads, cortical bone, lamina dura, adjacent roots, sinus floor, mandibular canal, radiopaque restorative materials, and areas of anatomical superimposition or image artifact were excluded. When the predefined ROI could not be reproducibly placed within trabecular bone without including these structures, that region was not measured. This rule was applied to both baseline and follow-up images to reduce measurement variability related to panoramic distortion and superimposition.

The rectangular 25 × 50 pixel configuration for mesial and distal ROIs was selected because it allowed sampling of the narrow peri-implant trabecular zone along the implant surface while minimizing inclusion of the implant margin, adjacent root structures, or cortical borders. The 50 × 50 pixel apical ROI was selected to sample the trabecular area immediately apical to the implant apex, where a broader trabecular compartment was usually available. These ROI dimensions were kept fixed to obtain comparable measurements across patients and time points and to provide sufficient trabecular information for box-counting analysis. This approach was selected with reference to previous dental fractal analysis studies using fixed ROI dimensions and to evidence indicating that ROI size, configuration, and placement may influence FD measurements [15,16].

**Fig 2:**
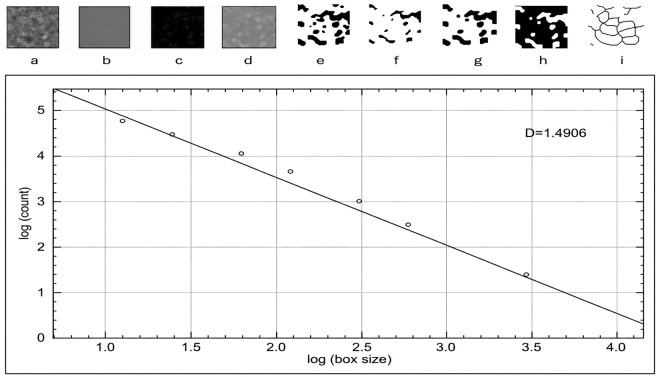
Workflow of fractal analysis applied to a region of interest (ROI) on a dental radiograph. **(a)** Duplicated image from the original radiograph; **(b)** Gaussian blur applied; (c) subtraction of the blurred image; (d) addition of a constant grayscale value (128); (e) binarization; (f) erosion; (g) dilation; (h) inversion; and (i) skeletonization. The log–log plot of the box-counting method illustrates the calculation of the fractal dimension (D = 1.4906).

Selected ROIs were cropped and duplicated for processing. A Gaussian blur with a 35-pixel radius was applied to the duplicate image, which was then subtracted from the original image to generate a high-pass filtered image. A constant grayscale value of 128 was added, followed by binarization to isolate trabecular structures. Image processing included “Erode,” “Dilate,” “Invert,” and “Skeletonize” operations. Fractal dimension (FD) was calculated using the box-counting method with grid sizes ranging from 2 to 64 pixels. The slope of the log–log plot of box size versus box count was accepted as the FD value (Fig 2). The same processing protocol was applied to all ROIs at baseline and at the 3-month follow-up.

### Statistical analysis

Statistical analyses were conducted using IBM SPSS Statistics (v27.0; IBM Corp., Armonk, NY, USA). Normality of continuous variables was assessed using the Shapiro–Wilk test. Paired sample t-tests were used to compare baseline and 3-month values within groups, while independent sample t-tests evaluated gender differences. If normality assumptions were not met, non-parametric alternatives (Wilcoxon signed-rank test and Mann–Whitney U test) were applied. Continuous data were presented as mean ± standard deviation (SD), and categorical variables as frequency (n) and percentage (%). A p-value < 0.05 was considered statistically significant. The primary outcome variable was defined as the change in mean peri-implant fractal dimension (FD), calculated as the average of the mesial and distal FD values between baseline and 3 months. A post hoc power analysis was performed for this primary outcome. Based on the observed effect size (Cohen’s dz = 1.29), the final sample size of 60 implants provided a statistical power of >0.99 at a two-sided alpha level of 0.05.

## Results

A total of sixty implants placed in sixty patients were included in the study, comprising thirty-six females and twenty-four males. For each implant, three regions of interest (ROIs), mesial, distal, and apical, were analyzed, resulting in a total of 360 ROIs across both the baseline and 3-month follow-up radiographs. The mean age of the female participants was 38.50 ± 12.31 years, while that of the male participants was 39.20 ± 11.40 years. The overall mean age of the study population was 38.80 ± 11.77 years, with no statistically significant difference between genders (p = 0.803).

When the fractal dimensions (FD) of the mesial, distal, and apical regions were examined for all implants, statistically significant increases were observed between baseline and the 3-month follow-up. The mean mesial FD increased from 1.214 ± 0.080 to 1.277 ± 0.058 (p < 0.001), the distal FD rose from 1.235 ± 0.071 to 1.287 ± 0.051 (p < 0.001), and the apical FD increased from 1.362 ± 0.048 to 1.383 ± 0.047 (p < 0.001). These findings indicate measurable early peri-implant trabecular structural changes during the 3-month unloaded healing period following implant placement. For the primary outcome, the mean peri-implant FD increased from 1.225 ± 0.059 at baseline to 1.282 ± 0.042 at 3 months, with a mean change of 0.057 (95% CI: 0.046 to 0.069; p < 0.001) (Table 1).

**Table 1:** Comparison of fractal dimension values at baseline and 3 months after implant placement.

Parameters	Pre-treatment (baseline) (Mean ± SD)	Post-treatment (3 months) (Mean ± SD)	p-value
**Mesial FD**	1.214 ± 0.080	1.277 ± 0.058	**< 0.001**
**Distal FD**	1.235 ± 0.071	1.287 ± 0.051	**< 0.001**
**Apical FD**	1.362 ± 0.048	1.383 ± 0.047	**< 0.001**
**Mean peri-implant FD**	1.225 ± 0.059	1.282 ± 0.042	**< 0.001**

FD: Fractal Dimension; Mean peri-implant FD: Mean peri-implant fractal dimension, calculated as the average of mesial and distal FD values.

Bold p values indicate statistically significant differences between baseline and 3-month measurements.

In secondary exploratory analyses, no statistically significant changes were identified in probing pocket depth (PPD), plaque index (PI), or bleeding on probing (BOP), with p-values of 0.792, 0.103, and 0.374, respectively. However, the gingival index (GI) showed a significant decrease from 2.00 ± 0.57 preoperatively to 1.61 ± 0.51 postoperatively (p < 0.001), whereas morphometric assessments of mandibular cortical width (MCW), mental length (ML), and the panoramic mandibular index (PMI) revealed no significant differences between baseline and 3-month measurements in either the right or left regions (p > 0.05), suggesting that cortical bone parameters remained stable during the early follow-up period (Table 2).

**Table 2:** Comparison of periodontal and morphometric parameters before and after implant placement.

Parameters	Pre-treatment (baseline) (Mean ± SD)	Post-treatment (3 months) (Mean ± SD)	p-value
**PPD (mm)**	2.54 ± 0.35	2.54 ± 0.34	0.792
**PI**	1.73 ± 0.46	1.88 ± 0.62	0.103
**GI**	2.00 ± 0.57	1.61 ± 0.51	**< 0.001**
**BOP (%)**	56.11 ± 26.28	60.60 ± 31.44	0.374
**MCW (left region)**	2.93 ± 0.84	2.82 ± 0.58	0.151
**MCW (right region)**	2.90 ± 0.90	2.82 ± 0.63	0.332
**Mental Length (left region)**	7.96 ± 1.22	7.97 ± 1.28	0.919
**Mental Length (right region)**	7.93 ± 1.51	8.04 ± 1.41	0.401
**PMI (left region)**	0.37 ± 0.10	0.36 ± 0.08	0.168
**PMI (right region)**	0.37 ± 0.10	0.36 ± 0.09	0.162

PPD: Probing Pocket Depth; PI: Plaque Index; GI: Gingival Index; BOP: Bleeding on Probing; MCW: Mandibular Cortical Width; PMI: Panoramic Mandibular Index.

Bold p values indicate statistically significant differences between baseline and 3-month measurements.

Comparisons between female and male participants showed no statistically significant gender-related differences in FD values or periodontal parameters at either time point (p > 0.05). The only exception was the plaque index, which was significantly higher among females at baseline (p = 0.025), although this difference was not present at 3 months (p = 0.738). With respect to morphometric indices, mental length values were consistently greater in males than in females at both baseline and 3 months (p < 0.05), whereas MCW and PMI did not differ significantly between genders (p > 0.05) (Table 3).

**Table 3:** Comparison of fractal dimension values, periodontal parameters, and morphometric indices between female and male patients at baseline and 3 months.

Parameters	Female/Pre-treatment (baseline) (Mean ± SD)	Male/Pre-treatment (baseline) (Mean ± SD)	p-value	Female/Post-treatment (3 months) (Mean ± SD)	Male/Post-treatment (3 months) (Mean ± SD)	p-value
**Mesial FD**	1.210 ± 0.079	1.219 ± 0.084	0.690	1.279 ± 0.058	1.274 ± 0.060	0.774
**Distal FD**	1.224 ± 0.078	1.251 ± 0.058	0.154	1.282 ± 0.051	1.295 ± 0.051	0.317
**Apical FD**	1.359 ± 0.048	1.368 ± 0.049	0.470	1.388 ± 0.044	1.374 ± 0.050	0.244
**Mean peri-implant FD**	1.217 ± 0.061	1.235 ± 0.055	0.258	1.280 ± 0.040	1.285 ± 0.045	0.683
**PPD**	2.52 ± 0.36	2.56 ± 0.33	0.651	2.52 ± 0.36	2.58 ± 0.32	0.424
**PI**	1.84 ± 0.49	1.55 ± 0.34	**0.025**	1.87 ± 0.61	1.90 ± 0.64	0.738
**GI**	2.06 ± 0.58	1.91 ± 0.55	0.345	1.63 ± 0.47	1.56 ± 0.56	0.938
**BOP (%)**	57.95 ± 25.77	53.34 ± 27.33	0.256	62.33 ± 32.63	58.00 ± 30.06	0.747
**MCW (left region)**	2.76 ± 0.68	3.19 ± 1.01	0.106	2.77 ± 0.56	2.90 ± 0.60	0.729
**MCW (right region)**	2.78 ± 0.59	3.09 ± 1.23	0.774	2.73 ± 0.58	2.95 ± 0.69	0.428
**Mental Length (left region)**	7.51 ± 1.12	8.63 ± 1.07	**< 0.001**	7.54 ± 1.08	8.61 ± 1.30	**0.002**
**Mental Length (right region)**	7.61 ± 1.50	8.42 ± 1.43	**0.040**	7.63 ± 1.32	8.66 ± 1.35	**0.006**
**PMI (left region)**	0.37 ± 0.09	0.37 ± 0.11	0.662	0.37 ± 0.09	0.34 ± 0.08	0.156
**PMI (right region)**	0.38 ± 0.09	0.37 ± 0.13	0.390	0.37 ± 0.10	0.34 ± 0.08	0.502

FD: Fractal Dimension; Mean peri-implant FD: Mean peri-implant fractal dimension, calculated as the average of mesial and distal FD values; PPD: Probing Pocket Depth; PI: Plaque Index; GI: Gingival Index; BOP: Bleeding on Probing; MCW: Mandibular Cortical Width; PMI: Panoramic Mandibular Index.

P values were calculated using independent samples t-test or Mann–Whitney U test, as appropriate. Bold p values indicate statistically significant differences between female and male patients.

Further subgroup analyses showed no significant associations between implant localization or implant length and the measured FD or morphometric parameters at either baseline or 3 months (p > 0.05). When the data were stratified according to baseline periodontal status, a significant difference was observed for distal FD at baseline (p = 0.002), whereas this difference was no longer significant at 3 months (p = 0.133). Mental length in the left region also differed significantly between the gingivitis and periodontitis groups at both baseline and 3 months (p = 0.016 and p = 0.038, respectively). No significant differences were identified for mesial FD, apical FD, MCW, or PMI according to periodontal status (p > 0.05) (Table 4).

**Table 4:** Exploratory subgroup comparisons of fractal dimension and morphometric parameters according to baseline periodontal status, implant localization, and implant length.

Parameters	Periodontal status (Gingivitis (n = 49)/ Periodontitis (n = 11)) Baseline	Periodontal status (Gingivitis (n = 49)/ Periodontitis (n = 11)) 3 months	Localization (Maxilla (n = 29)/ Mandible (n = 31)) Baseline	Localization (Maxilla (n = 29)/ Mandible (n = 31)) 3 months	Implant length (Short (n = 44)/ Long (n = 16)) Baseline	Implant length (Short (n = 44)/ Long (n = 16)) 3 months
**Mesial FD**	0.120	0.922	0.178	0.276	0.817	0.396
**Distal FD**	**0.002**	0.133	0.426	0.626	0.393	0.714
**Apical FD**	0.233	0.094	0.418	0.776	0.156	0.384
**MCW (left region)**	0.157	0.156	0.860	0.946	0.824	0.697
**MCW (right region)**	0.290	0.527	0.468	0.520	0.745	0.574
**Mental Length (left region)**	**0.016**	**0.038**	0.838	0.932	0.326	0.202
**Mental Length (right region)**	0.393	0.057	0.694	0.198	0.254	0.215
**PMI (left region)**	0.965	0.967	0.890	0.961	0.392	0.654
**PMI (right region)**	0.474	0.558	0.242	0.524	0.724	0.770

FD: Fractal Dimension; MCW: Mandibular Cortical Width; PMI: Panoramic Mandibular Index.

Note: This table presents exploratory subgroup comparisons of FD and morphometric parameters according to baseline periodontal status (gingivitis/periodontitis), implant localization (maxilla/mandible), and implant length (short/long). The p-values for each parameter are shown separately for baseline and 3-month measurements.

P values were calculated using independent samples t-test or Mann–Whitney U test, as appropriate. Bold p values indicate statistically significant subgroup differences.

## Discussion

This study aimed to evaluate early trabecular changes around dental implants by employing panoramic morphometric indices and FA on radiographs obtained before implant placement and at 3 months postoperatively. The results revealed significant postoperative increases in FD values, particularly in the mesial, distal, and apical regions, suggesting measurable early peri-implant trabecular structural changes during the 3-month unloaded healing period. These findings are consistent with the concept that early peri-implant structural changes may first be reflected in the trabecular compartment before more evident cortical adaptations become detectable.

Panoramic morphometric indices such as MCW and PMI did not show significant alterations between baseline and postoperative evaluations. This suggests that FD-based trabecular structural changes may be detectable before changes in cortical morphometric indices become evident, highlighting the potential sensitivity of FA compared with conventional panoramic morphometric indices during early radiographic follow-up. Previous studies have similarly reported that early peri-implant structural changes may initially be reflected in the trabecular compartment, whereas cortical bone changes require longer follow-up periods to become radiographically evident [17,18]. In addition, MCW, ML, and PMI are based on cortical and mental foramen-related measurements rather than on the local peri-implant trabecular ROI itself. Therefore, these indices may be less sensitive to short-term, site-specific trabecular structural changes during the early unloaded healing period and may require longer observation to reflect more established structural adaptation.

The initial null hypothesis of this study proposed that no significant changes would occur in FA or morphometric indices during the 3-month healing period. Our results partially rejected this hypothesis: FD values increased significantly across all regions, while cortical indices such as MCW and PMI remained stable. These findings suggest that FA may be capable of detecting early trabecular structural changes during the first 3 months after implant placement, whereas conventional panoramic morphometric indices remained unchanged in this cohort. In addition, the primary outcome analysis, based on mean peri-implant FD calculated from the mesial and distal regions, also demonstrated a significant increase over the follow-up period, providing additional support for the interpretation of early peri-implant trabecular change within the limitations of the present study. Our observations are in line with several investigations that reported postoperative increases in FD and associated these changes with new trabecular organization and improved bone complexity [10,19,20]. However, discrepancies in the literature remain, as some studies have reported no significant FD changes or limited prognostic utility of fractal metrics, likely due to variations in imaging modalities, ROI selection, surgical protocols, study endpoints, or follow-up durations [21–23]. Recent evidence has also shown that the association between panoramic FD measurements and implant stability may depend on ROI configuration, further underscoring the importance of standardized ROI selection in peri-implant radiographic analysis [24]. The dynamic nature of bone remodeling further underscores the importance of longitudinal assessment. For instance, Heo et al. documented a transient reduction in FD after orthognathic surgery followed by recovery, reflecting the non-linear progression of bone healing [25].

Clinical periodontal parameters were also evaluated as secondary exploratory variables. In the present study, GI showed a significant decrease over the follow-up period, whereas PPD, PI, and BOP did not change significantly. Although these findings may indicate some improvement in the local clinical periodontal environment during early healing, they should be interpreted cautiously, as the measurements were obtained from natural teeth in the same quadrant rather than directly from the implant sites. Because only GI changed while the other periodontal parameters remained stable, these findings do not indicate a consistent local periodontal effect that could directly account for the observed increase in FD values. The isolated decrease in GI is more likely to reflect a general reduction in gingival inflammatory status during the early follow-up period rather than a direct determinant of peri-implant trabecular structural changes. Similar peri-implant studies have shown that plaque- and inflammation-related clinical indices and radiographic bone assessments represent complementary but distinct dimensions of evaluation [19,26]. Therefore, the FA findings represent the main radiographic evidence of early structural bone change in this study, while the periodontal parameters provide supportive clinical context. Accordingly, no direct ROI-level relationship between the periodontal parameters and peri-implant FD values should be assumed in the present study.

From a clinical standpoint, these findings suggest that FA may have potential as a practical adjunctive tool for early radiographic follow-up. The clinical value of detecting such early changes lies in identifying radiographic trabecular structural changes during the early follow-up period, even when conventional morphometric indices remain unchanged. This may support closer radiographic monitoring and more cautious interpretation of early healing patterns, particularly when additional mechanical stability assessment is not available. Unlike cone-beam computed tomography (CBCT), which involves higher radiation exposure and variable accessibility, or resonance frequency analysis (RFA) and implant stability quotient (ISQ), which require additional equipment that may not be available in every clinical setting, FA can be performed on panoramic radiographs obtained when clinically indicated during follow-up. This makes FA an accessible, low-radiation, and non-invasive method that can provide supplementary information about early peri-implant bone dynamics without the need for specialized devices. However, it is important to emphasize that FA reflects radiographic changes in trabecular microarchitecture rather than direct evidence of osseointegration. Thus, FA findings should be interpreted as early indicators of radiographic trabecular structural change, not as definitive proof that osseointegration has occurred. This cautious interpretation is also supported by recent evidence suggesting that, although fractal analysis may provide non-invasive insight into trabecular bone architecture, its standalone use does not appear sufficiently reliable for predicting early implant failure [23]. Mechanical stability measurements such as RFA and ISQ remain the established objective tools for confirming osseointegration; therefore, FA should be regarded as a complementary method that enhances radiographic follow-up rather than a standalone diagnostic indicator.

From a demographic perspective, no significant age- or gender-related differences in FD values were observed, although these findings should be interpreted cautiously in view of the retrospective design, modest sample size, unequal gender distribution, and short 3-month follow-up period of the present study. This interpretation is supported by recent panoramic radiography-based evidence showing no statistically significant effect of age or gender on FD values, despite the presence of localized trabecular alterations in the region of interest [27]. Previous reports have emphasized the potential effects of systemic conditions such as osteoporosis, hormonal status, or long-term metabolic changes on implant prognosis, but such influences may not manifest within the early 3-month period investigated here [28–30].

This study has several limitations. The retrospective design and modest sample size may limit the generalizability of the findings. Follow-up was limited to the early 3-month period, which prevents conclusions about long-term peri-implant trabecular structural changes. Furthermore, only two-dimensional panoramic radiographs were used, which limited volumetric assessment compared to cone beam computed tomography (CBCT) or micro-CT. Although ROI placement was standardized using fixed dimensions and reproducible radiographic landmarks, panoramic radiographs are inherently affected by magnification, geometric distortion, and anatomical superimposition. Therefore, minor variations in ROI positioning between baseline and follow-up images cannot be completely excluded, particularly when small peri-implant ROIs are used. This limitation should be considered when interpreting subtle changes in FD values. Although cases were not excluded solely on the basis of clinically detectable crestal bone loss, radiographs were excluded when crestal bone loss, peri-implant radiolucency, cortical disruption, or related radiographic changes prevented reproducible ROI placement. This approach may have reduced measurement variability, but it may also limit the generalizability of the findings to implants with radiographically stable peri-implant bone conditions. In addition, clinical stability measurements such as resonance frequency analysis (RFA) or implant stability quotient (ISQ), which could provide complementary evidence of osseointegration, were also not available. Another limitation was the absence of clinical attachment loss (CAL) values, an important parameter in periodontal diagnosis, which were not consistently recorded in the retrospective archive and therefore could not be reliably analyzed. In addition, because the periodontal measurements were obtained from natural teeth rather than implant sites, any relationship between these parameters and peri-implant bone changes should be interpreted cautiously. Finally, the use of a single implant system limited extrapolation to other implant designs.

Despite these limitations, the study presents several strengths. The use of a homogeneous single-tooth sample minimized confounding influences from multiple missing teeth or adjacent implants. All implants were placed by the same experienced surgeon using the BIL® Dental Implant System (BIL Implant, Istanbul, Türkiye) under standardized protocols, reducing variability related to operator technique or implant design. Radiographs were acquired with a uniform protocol by the same device and technician, ensuring methodological consistency. The sample size of sixty implants is larger than that of many previous FA-based studies, enhancing statistical robustness. Moreover, the combined use of FA and morphometric indices allowed simultaneous evaluation of trabecular and cortical compartments, providing a more comprehensive perspective on early peri-implant radiographic bone assessment. Importantly, ROI dimensions were standardized in accordance with published FA protocols, and TIFF image format was employed to avoid grayscale data loss, thereby improving methodological reliability.

## Conclusions

FA demonstrated detectable early trabecular changes around implants during the first 3 months after implant placement, highlighting its ability to detect subtle trabecular structural changes that were not detected by conventional morphometric indices in this early period. Although these early FA findings cannot independently confirm osseointegration, they offer valuable supplementary information for non-invasive radiographic follow-up. The clinical periodontal parameters evaluated in this study should be interpreted as supportive contextual findings rather than direct measures of peri-implant healing. The significant increase observed in the primary outcome, defined as mean peri-implant FD, suggests that FA may be useful for monitoring early peri-implant trabecular changes, although this interpretation should be considered within the limitations of the present retrospective study. Future prospective studies with longer observation periods and mechanical stability assessments such as ISQ are needed to further clarify the clinical utility of FA in early peri-implant radiographic assessment.

## Supporting information

S1 Data De-identified baseline and 3-month clinical and radiographic measurement data underlying the findings of this study. (XLSX)
