# Supplementary material for: Evaluation of early trabecular changes around implants using fractal analysis and panoramic indices
Source: PLoS One. 2026 Jul 1;21(7):e0352984. doi: 10.1371/journal.pone.0352984 (PMC13322550; doi:10.1371/journal.pone.0352984)
Supplement: Attachment — Submitted filename: Response to Reviewers.docx [file pone.0352984.s003.docx]

**Response to Academic Editor**

**Academic Editor - Comment 1:** The study rationale should be clarified, particularly the role and contribution of clinical periodontal parameters in relation to peri-implant bone healing.

**Response:** Thank you for this important comment. We have revised the manuscript to clarify the study rationale and to better define the role of the clinical periodontal parameters. In the revised Introduction, we emphasized that the primary aim of the study was to evaluate early peri-implant alveolar bone changes by comparing fractal dimension (FD) values obtained from panoramic radiographs at baseline and at 3 months postoperatively. We also clarified that the analyses involving panoramic morphometric indices and clinical periodontal parameters were secondary and exploratory in nature. In the Materials and Methods section, we specified that the periodontal parameters were obtained from natural teeth in the same quadrant and were evaluated as secondary clinical variables for exploratory comparison with the radiographic findings. In addition, the Discussion and Conclusion sections were revised to present these periodontal findings more cautiously, as supportive clinical context rather than direct indicators of peri-implant bone healing. These revisions were made to ensure a clearer distinction between the primary radiographic objective of the study and the supplementary role of the clinical periodontal parameters. **(Introduction-Paragraphs 1,3,4 and 5; Materials and Methods-Paragraph 3; Results-Paragraphs 2,3 and 5; Discussion-Paragraphs 3,4,5 and 6; Conclusions)**

**Academic Editor - Comment 2:** The inconsistency in ethics approval information must be corrected, and all related details (approval number, committee name, and date) should be fully consistent throughout the manuscript and submission system.

**Response:** Thank you for pointing this out. We carefully reviewed the ethics approval document and corrected the manuscript accordingly. The correct ethics approval information is now reported consistently as Necmettin Erbakan University Faculty of Dentistry Non-Drug and Non-Medical Device Research Ethics Committee, Approval No. 2025/639, dated 31.07.2025. We also revised the submission metadata to ensure that the committee’s name, approval number, and date are fully consistent across all parts of the submission. In addition, in accordance with the journal’s requirement, the ethics statement was retained only in the Materials and Methods section and removed from the separate Ethics statement section. **(Materials and Methods-Paragraph 1)**

**Academic Editor - Comment 3:** The Materials and Methods section requires substantial clarification, including power analysis, participant characteristics (e.g., age range), ROI selection and reproducibility, and clear definitions of all subgroups (implant region, implant length, periodontal status, etc.).

**Response:** Thank you for this helpful comment. We substantially revised the Materials and Methods section to improve methodological clarity and transparency. Specifically, we added the age range of the study population and clarified the participant characteristics. We also defined the primary outcome variable as the change in mean peri-implant fractal dimension (FD), calculated as the average of the mesial and distal FD values between baseline and 3 months, and included a post hoc power analysis based on this outcome. In addition, we expanded the description of ROI selection by specifying the positioning of the mesial, distal, and apical ROIs, the reference points used for standardization, and the rationale for the selected ROI dimensions. We further clarified the subgroup definitions used in the analyses, including implant localization, implant length, and baseline periodontal status, and we specified that morphometric measurements were evaluated separately on the right and left sides because of possible side-related variation on panoramic radiographs. To improve consistency between the Methods, Results, and data presentation, we also revised and reorganized the tables so that the primary FD analysis, secondary periodontal and morphometric analyses, gender-based comparisons, and exploratory subgroup analyses are now presented more clearly and in alignment with the revised analytical framework. **(Materials and Methods-Paragraphs 2,3,8,9 and 10; Results-Paragraph 2; Tables 1-4)**

**Academic Editor - Comment 4:** The presentation of results should be improved. Tables and figures must be revised for clarity, consistency, and accuracy. In particular, Table 3 should be restructured, and figure quality should be significantly improved.

**Response:** Thank you for this helpful comment. We revised the presentation of the Results section to improve clarity, consistency, and accuracy. The tables were reorganized to better reflect the analytical structure of the study. Specifically, the primary FD analysis, the secondary periodontal and morphometric comparisons, the gender-based comparisons, and the exploratory subgroup analyses are now presented in separate tables with revised titles, footnotes, and table numbering. The former subgroup table was restructured to improve readability and to ensure full consistency between its content, heading, and explanatory note. We also reviewed the tables for numerical consistency with the revised dataset and updated the Results text accordingly. In addition, the figures were revised to improve visual clarity and to better illustrate the anatomical reference points and measured parameters. **(Results-Paragraphs 2-5; Tables 1-4; Figure 1)**

**Academic Editor - Comment 5:** The discussion should be revised to provide a more cautious interpretation of the findings, considering the retrospective design, sample size, and follow-up period.

**Response:** Thank you for this important comment. We revised the Discussion and Conclusion sections to provide a more cautious interpretation of the findings. Specifically, we tempered the clinical interpretation of the observed FD changes and avoided overstatement regarding the role of fractal analysis in implant healing. We emphasized that the study was retrospective in design, included a modest sample size, and was limited to a 3-month follow-up period, which precludes definitive conclusions regarding long-term bone remodeling or osseointegration. We also clarified that fractal analysis should be interpreted as a complementary radiographic method reflecting early trabecular reorganization rather than as a standalone or confirmatory indicator of osseointegration. In addition, the limitations related to the study design, follow-up duration, and the contextual nature of the periodontal variables were made more explicit in the revised Discussion. The Conclusion section was also revised accordingly to maintain a more cautious and balanced interpretation. **(Results-Paragraphs 2 and 5; Discussion-Paragraphs 3,5,6 and 7; Conclusions)**

**Academic Editor - Comment 6:** Language and terminology should be carefully revised to correct spelling errors and ensure consistency throughout the manuscript.

**Response:** Thank you for this helpful comment. We carefully revised the manuscript for language, spelling, punctuation, and terminology consistency throughout the text, tables, and figure legends. Several typographical errors were corrected, and terminology was standardized across all sections of the manuscript. In particular, radiographic and morphometric terms, subgroup labels, and abbreviations were reviewed to ensure consistent usage in the main text, tables, and explanatory notes. We also corrected formatting and wording inconsistencies that arose during revision to improve the overall clarity and readability of the manuscript.

**Academic Editor - Comment 7:** The introduction could be improved by presenting the originality and clinical relevance of fractal analysis in a more balanced manner.

**Response:** Thank you for this valuable comment. We revised the Introduction to present the originality and clinical relevance of fractal analysis in a more balanced and cautious manner. Specifically, we reduced overly strong wording regarding the potential role of fractal analysis, clarified that its value in detecting early peri-implant trabecular changes on panoramic radiographs remains an area requiring further investigation, and emphasized the study’s focus on early radiographic assessment rather than definitive prediction of implant success. We also restructured the rationale of the study to better distinguish the primary radiographic objective from the secondary exploratory analyses. **(Introduction-Paragraphs 1-5)**

**Academic Editor - Comment 8:** Additional recent literature may be incorporated to support the interpretation of findings.

**Response:** Thank you for this helpful suggestion. We incorporated additional recent literature into the revised manuscript to strengthen and update the interpretation of our findings. The newly added references address peri-implant bone architecture, early trabecular remodeling, the interpretive limits of fractal analysis in implant-related outcomes, and the methodological importance of ROI configuration in panoramic radiographic assessment. These references were integrated into the Discussion section to provide a more current and balanced framework for interpreting the observed FD changes and to improve comparison with recent evidence. **(Discussion-Paragraphs 4 and 6; References 20,23 and 24)**

**Academic Editor - Comment 9:** Visual presentation of morphometric measurements (e.g., mandibular indices) could be enhanced for better reader understanding.

**Response:** Thank you for this helpful suggestion. We revised the visual presentation of the morphometric measurements to improve reader understanding. Figure 1 was updated to show the MCW measurement, the mental foramen reference point, and the peri-implant ROI locations more clearly, with improved image quality and clearer annotations. The figure legend was also revised accordingly to provide a more explicit explanation of the anatomical landmarks and measured parameters. **(Figure 1; Figure Legends)**

**Journal Requirements**

**Comment 1:** Please ensure that your manuscript meets PLOS ONE's style requirements, including those for file naming.

**Response:** Thank you for this helpful comment. We carefully revised the manuscript to align it more closely with PLOS ONE style requirements. Specifically, we updated the title and affiliation formatting, standardized section headings, placed figure captions and tables after their first citation in the text, and revised the supporting information section accordingly. We also checked the file naming and overall submission formatting to improve consistency with PLOS ONE requirements.

**Comment 2:** The ethical approval number(s) listed in the manuscript and/or submission metadata does not match the approval number on the ethical approval document you provided. Please ensure that all approval numbers are correct.

**Response:** Thank you for pointing this out. We carefully reviewed the ethics approval document and corrected the manuscript and submission metadata accordingly. The correct ethics approval information is now reported consistently throughout the submission as Necmettin Erbakan University Faculty of Dentistry Non-Drug and Non-Medical Device Research Ethics Committee, Approval No. 2025/639, dated 31.07.2025. We also checked the relevant sections of the manuscript to ensure full consistency in the committee name, approval number, and date. **(Materials and Methods-Paragraph 1)**

**Comment 3:** We note that there is identifying data in the Supporting Information file S1 File.xlsx. Due to the inclusion of these potentially identifying data, we have removed this file from your file inventory. Prior to sharing human research participant data, authors should consult with an ethics committee to ensure data are shared in accordance with participant consent and all applicable local laws. Please remove or anonymize all personal information, ensure that the data shared are in accordance with participant consent, and re-upload a fully anonymized data set. Please note that spreadsheet columns with personal information must be removed and not hidden, as all hidden columns will appear in the published file.

**Response:** Thank you for this important note. We re-examined the original supporting spreadsheet and prepared a fully anonymized replacement file. All potentially identifying information was removed from the dataset, and no identifying columns were retained in hidden form. The revised supporting file now includes only de-identified measurement data necessary to support the reported findings. We also confirmed that the shared dataset is consistent with participant privacy protection and the retrospective use of anonymized data. The corrected file has been re-uploaded as S1 Data. **(Supporting information-S1 Data)**

**Comment 4:** Your ethics statement should only appear in the Methods section of your manuscript. If your ethics statement is written in any section besides the Methods, please move it to the Methods section and delete it from any other section. Please ensure that your ethics statement is included in your manuscript, as the ethics statement entered into the online submission form will not be published alongside your manuscript.

**Response:** Thank you for this clarification. We revised the manuscript accordingly so that the ethics statement now appears only in the Materials and Methods section. Any duplicate ethics statement presented elsewhere in the manuscript was removed. We also confirmed that the full ethics approval information remains included in the manuscript text, in accordance with the journal’s requirements. **(Materials and Methods-Paragraph 1)**

**Comment 5:** Please include your Supporting Information captions at the end of the manuscript file. If any Supporting Information files are cited in the manuscript, please ensure that the file names and captions are presented consistently.

**Response:** Thank you for this clarification. We revised the manuscript accordingly and added the Supporting Information caption at the end of the manuscript file. We also reviewed the manuscript for consistency between the in-text citation, file label, and uploaded supporting file. The supporting dataset is now cited and labeled consistently as S1 Data throughout the revised submission. **(S1 Data; Supporting information)**

**Comment 6:** Please consider the reviewer and editor suggestions regarding additional relevant literature and revise the manuscript accordingly.

**Response:** Thank you for this note. We carefully reviewed the publications recommended in the reviewer comments and evaluated their relevance to the scope, methodology, and interpretation of the present study. Relevant works were incorporated into the revised manuscript where appropriate to strengthen the discussion and improve comparison with the literature. References that were not directly relevant to the objectives or methodological framework of the present study were not added. (**Discussion-Paragraphs 2,3,5 and 6; References 20,23,24 and 27)**

**Reviewers' comments**

**Reviewer 1 Comment 1:** In the introduction, although the main focus of the study is peri-implant bone healing, the rationale for including clinical periodontal parameters should be explained more clearly. In addition, if the statements regarding the value of fractal analysis in predicting implant success and the originality of the study are presented in a more cautious manner, the text will appear more balanced.

**Response:** Thank you for this valuable comment. We revised the Introduction to clarify the rationale for including clinical periodontal parameters and to present the role of fractal analysis in a more balanced and cautious manner. Specifically, we clarified that clinical periodontal parameters were included as secondary exploratory variables to provide additional clinical context for the local oral environment during early healing, whereas the primary focus of the study remained the radiographic assessment of peri-implant bone changes. We also softened the wording regarding the clinical value of fractal analysis and avoided overly strong statements concerning implant success prediction and the originality of the study. In the revised Introduction, the study aim is now presented more clearly as the evaluation of early peri-implant alveolar bone changes using FD values derived from panoramic radiographs, with morphometric indices and clinical periodontal parameters addressed as secondary exploratory analyses. **(Introduction-Paragraphs 1,3,4 and 5)**

**Reviewer 1 - Comment 2**: There is an inconsistency in the ethics approval information. In the ethics statement entered in the submission system, the ethics committee decision is reported as “2025/639” with the date “July 31, 2025,” whereas in the manuscript text it is stated as “2024/460.” This discrepancy should be corrected. The ethics approval number, committee name, and date should be fully consistent across all sections of the submission.

**Response:** Thank you for pointing this out. We carefully reviewed the ethics approval document and corrected the manuscript accordingly. The ethics approval information is now reported consistently as Necmettin Erbakan University Faculty of Dentistry Non-Drug and Non-Medical Device Research Ethics Committee, Approval No. 2025/639, dated 31.07.2025. We also checked the relevant submission information to ensure full consistency in the committee name, approval number, and date across all parts of the submission. **(Materials and Methods-Paragraph 1)**

**Reviewer 1 - Comment 3:** In the Materials and Methods section, the contribution of the periodontal parameters obtained from natural teeth to the study should be stated more clearly. Figure 1 could be improved by presenting the mandibular indices more clearly. A more explicit visualization of the anatomical reference points and measured parameters would help the reader better understand the morphometric assessment.

**Response:** Thank you for this valuable comment. We revised the Materials and Methods section to better define the contribution of the periodontal parameters obtained from natural teeth, clarifying that these variables were included as secondary exploratory clinical parameters to provide clinical context for the radiographic findings during the unloaded healing period. We also improved Figure 1 and its legend to present the morphometric measurements, anatomical reference point, and peri-implant ROI locations more explicitly, thereby facilitating clearer understanding of the mandibular indices and measurement approach. **(Materials and Methods-Paragraph 3; Figure 1; Figure Legends)**

**Reviewer 1 - Comment 4:** In Table 3, implant location and implant length are presented together; however, the table title and footnote are not fully consistent with the actual structure of the table. In addition, the footnote refers to “implant design” and “implant region,” although these variables are not clearly presented in the table itself. In its current form, the table is difficult to read and somewhat confusing. A clearer presentation would be preferable, ideally by separating these results into different tables and indicating more explicitly which comparison each p-value refers to.

**Response:** Thank you for this helpful comment. The former Table 3 has been reorganized and is now presented as Table 4 to improve clarity, consistency, and readability. The subgroup results were retained in a single exploratory comparison table, but the title, subgroup headings, and explanatory note were revised substantially to ensure consistency with the actual table structure. References to variables that were not presented in the table, including “implant design” and “implant region,” were removed. In addition, the revised table now indicates more explicitly which comparison each p-value refers to by separating subgroup type and time point (baseline or 3 months). The updated presentation provides a clearer and more consistent summary of the subgroup analyses. **(Table 4)**

**Reviewer 1 - Comment 5:** In the discussion section, expressing the comments regarding the clinical utility of fractal analysis and the limited influence of age/gender in a more cautious manner, taking into account the retrospective design, short follow-up period, and sample size of the study, could strengthen the discussion. It may also be useful to include recent literature supporting the use of fractal analysis on panoramic radiographs for the assessment of trabecular bone microarchitecture (e.g., doi: 10.1186/s12880-026-02193-7).

**Response:** Thank you for this valuable comment. We revised the Discussion and Conclusion sections to adopt a more cautious and balanced interpretation of the findings. Specifically, we tempered the statements regarding the potential clinical utility of fractal analysis and the limited effects of age and sex by emphasizing that these observations should be interpreted within the constraints of the retrospective design, modest sample size, unequal gender distribution, and relatively short 3-month follow-up period. We also clarified that fractal analysis should be regarded as a complementary radiographic method reflecting early trabecular reorganization rather than as a definitive or standalone indicator of implant prognosis or osseointegration. In addition, we incorporated recent literature into the Discussion, including the suggested study, to provide a more current context for the interpretation of the observed FD changes and to support the use of panoramic fractal analysis for the assessment of trabecular bone microarchitecture. **(Discussion-Paragraphs 3,5,6 and 7; Conclusions; Reference 27)**

**Reviewer 1 - Comment 6:** In the conclusion section, it may be helpful to express the comparison with clinical parameters more cautiously, since these measurements were obtained not from the implant sites but from natural teeth in the same quadrant.

**Response:** Thank you for this valuable comment. We revised the Conclusion section to express the comparison with the clinical parameters more cautiously. Specifically, we clarified that the periodontal measurements were obtained from natural teeth in the same quadrant rather than from the implant sites and therefore should not be interpreted as direct measures of peri-implant healing. In the revised Conclusion, these variables are now described as supportive contextual findings, while the main interpretation of early healing is based on the radiographic findings derived from fractal analysis**. (Conclusions)**

**Reviewer 1 - Comment 7:** Some language and spelling issues should also be corrected. For example, the term “fractal dimesion” is misspelled in the abstract. Similarly, the expressions “Panoramic Mandibular Index” and “Panoramic Mental Index” are not used consistently across the tables. Although the manuscript is generally understandable, it would benefit from careful language editing in terms of terminology and formal presentation.

**Response:** Thank you for this helpful comment. We carefully revised the manuscript for language, spelling, punctuation, and terminology consistency throughout the text, tables, and figure legends. The misspelling of “fractal dimesion” in the abstract was corrected to “fractal dimension,” and inconsistent terminology related to the mandibular indices was standardized throughout the manuscript. In particular, expressions such as “Panoramic Mandibular Index” and related abbreviations were reviewed and used consistently across the text and tables. We also corrected additional typographical and formatting inconsistencies to improve the formal presentation and overall readability of the manuscript. **(Manuscript; Tables 2-4)**

**Reviewer 2 - Abstract Comment 1:** There are spelling errors in the abstract section. It should be reviewed again. For example, (fractal dimesion → dimension).

**Response:** Thank you for this helpful comment. We carefully reviewed the abstract for spelling and language consistency and corrected the typographical error identified by the reviewer. Specifically, the term “fractal dimesion” was corrected to “fractal dimension.” The abstract was also rechecked to improve overall formal presentation and consistency of terminology. **(Abstract)**

**Reviewer 2 - Introduction Comment 1:** “have gained widespread acceptance” → can be revised as “are widely accepted”.

**Response:** Thank you for this helpful suggestion. We revised the opening sentence of the Introduction accordingly. The previous wording was simplified, and the statement now reads that dental implant treatments *are widely accepted* for the rehabilitation of partially or completely edentulous jaws. **(Introduction-Paragraph 1)**

**Reviewer 2 - Introduction Comment 2:** The reason for preferring 3 months in the early-period evaluation should be explained in more detail.

**Response:** Thank you for this helpful comment. We revised the Introduction to explain more clearly why the 3-month interval was selected for early-period evaluation. The revised text now emphasizes that this period represents an early healing phase in which trabecular reorganization may occur before more evident cortical or morphometric changes become apparent, thereby providing a relevant time point for assessing early peri-implant bone remodeling. **(Introduction-Paragraph 4)**

**Reviewer 2 - Introduction Comment 3:** An example of an early-period study should be added, and the contribution of the present study to early-period research should be emphasized.

**Response:** Thank you for this helpful comment. We revised the Introduction accordingly. In the revised text, we added a more explicit example of early-period peri-implant research using fractal analysis and clarified that the present study specifically focuses on the unloaded 3-month healing period. We also emphasized the contribution of the study more clearly by stating that it aims to add to the limited evidence on early peri-implant trabecular changes assessed on panoramic radiographs. **(Introduction-Paragraph 4; Reference 11)**

**Reviewer 2 - Introduction Comment 4:** The effect of periodontal parameters in the hypothesis remains unclear. Further clarification is required.

**Response:** Thank you for this helpful comment. We revised the final paragraph of the Introduction to clarify the role of the periodontal parameters in the study design and hypothesis structure. Specifically, we stated more clearly that clinical periodontal parameters were evaluated only as secondary exploratory variables, whereas the primary aim and formal null hypothesis of the study were limited to the comparison of peri-implant FD values between baseline and 3 months. To avoid ambiguity, we also added that no formal hypothesis was established for the secondary exploratory analyses involving panoramic morphometric indices and clinical periodontal parameters. **(Introduction-Paragraph 5)**

**Reviewer 2 - Introduction Comment 5:** In line 66, the expression radiographs.1 is incorrect.

**Response:** Thank you for pointing this out. We corrected this typographical/formatting error in the Introduction and reviewed the surrounding text for punctuation and reference-placement consistency. The sentence has been revised accordingly in the manuscript. **(Introduction-Paragraph 3)**

**Reviewer 2 - Introduction Comment 6:** In line 69, an em dash (—) should be used: “for implant success—particularly in the early healing phase—requires further investigation.”

**Response:** Thank you for this helpful comment. We revised the relevant sentence in the Introduction accordingly and corrected the punctuation/style as suggested by the reviewer. **(Introduction-Paragraph 3)**

**Reviewer 2 - Materials and Methods Comment 1:** The ethics approval numbers do not match those in the Ethics Statement section.

**Response:** Thank you for pointing this out. We carefully reviewed the ethics approval information and corrected the manuscript accordingly. The ethics approval is now reported consistently in the Materials and Methods section as Necmettin Erbakan University Faculty of Dentistry Non-Drug and Non-Medical Device Research Ethics Committee (Approval No. 2025/639; 31.07.2025). We also checked the corresponding ethics information in the submission to ensure consistency of the committee name, approval number, and date across all sections of the revised submission. **(Materials and Methods-Paragraph 1)**

**Reviewer 2 - Materials and Methods Comment 2:** Power analysis is missing.

**Response:** Thank you for this comment. We revised the Statistical Analysis section to address this point. Specifically, we defined the primary outcome as the change in mean peri-implant fractal dimension (FD), calculated as the average of the mesial and distal FD values between baseline and 3 months, and added a post hoc power analysis based on this outcome. The revised manuscript now reports the observed effect size and the corresponding statistical power of the final sample. **(Materials and Methods-Paragraph 12)**

**Reviewer 2 - Materials and Methods Comment 3:** The age range of participants has not been specified.

**Response:** Thank you for this helpful comment. We revised the Materials and Methods section to include the age range of the study population. The participant characteristics are now reported as 24 males and 36 females, with an age range of 21–74 years. **(Materials and Methods-Paragraph 2)**

**Reviewer 2 - Materials and Methods Comment 4:** The reproducibility of ROI selection is unclear. Reference points used for selecting mesial-distal and apical regions could be added.

**Response:** Thank you for this helpful comment. We revised the Fractal Analysis Procedure section to clarify the reproducibility of ROI selection and to specify the reference points used for ROI placement. In the revised manuscript, we now state that the mesial and distal ROIs were positioned parallel to the implant surface along the peri-implant trabecular bone, whereas the apical ROI was positioned immediately apical to the implant tip. We also clarified that ROI placement was standardized using the implant outline, implant apex, and adjacent root contours as reference points, thereby improving the transparency and reproducibility of the method. **(Materials and Methods-Paragraph 10)**

**Reviewer 2 - Materials and Methods Comment 5:** The rationale for ROI size should be explained.

**Response:** Thank you for this helpful comment. We revised the Fractal Analysis Procedure section to explain the rationale for the selected ROI dimensions more clearly. In the revised manuscript, we now state that these ROI sizes were used to obtain comparable peri-implant trabecular areas while minimizing the inclusion of cortical borders, implant margins, and overlapping anatomical structures. We also added methodological references to support the standardization of ROI configuration in panoramic fractal analysis**. (Materials and Methods-Paragraph 10; References 15 and 16)**

**Reviewer 2 - Materials and Methods Comment 6:** Implant placement regions (anterior/posterior) should be included. The region may influence analysis results.

**Response:** Thank you for this helpful comment. We clarified the implant placement region in the Materials and Methods section. Specifically, only posterior single-tooth implant sites were included in the present study, and this has now been stated explicitly in the revised manuscript. Therefore, an additional anterior/posterior subgroup analysis was not applicable. Restricting the sample to posterior sites also helped reduce anatomical heterogeneity in the radiographic evaluation. **(Materials and Methods-Paragraph 2)**

**Reviewer 2 - Materials and Methods Comment 7:** The use of ‘-’ vs. ‘—’ (em dash) and commas should be reviewed.

**Response:** Thank you for this helpful comment. We carefully reviewed the manuscript for consistency in punctuation and formal style. In particular, the use of hyphens, em dashes, and commas was standardized throughout the revised manuscript, including the Materials and Methods section, to improve readability and presentation. **(Materials and Methods-Paragraphs 9-11)**

**Reviewer 2 - Results Comment 1:** The relationship between periodontal measurements and the region of interest is weak. The relationship between periodontal parameters and FA should be explained.

**Response:** Thank you for this important comment. We clarified this point throughout the revised manuscript. In the Materials and Methods section, we now state more explicitly that the periodontal measurements were obtained from natural teeth in the same quadrant, not from the implant sites, and that they were included as secondary exploratory clinical parameters intended to provide clinical context for the radiographic findings rather than direct measures of peri-implant healing. In the Results section, these variables are presented as secondary exploratory findings, and only limited change was observed, with a significant decrease in gingival index but no significant differences in the other periodontal parameters. In the Discussion, we further clarified that the FA findings constitute the main radiographic evidence of early structural bone change in the present study, whereas the periodontal parameters should be interpreted as supportive contextual findings and not as direct ROI-level correlates of peri-implant FD values. **(Materials and Methods-Paragraph 3; Results-Paragraph 3; Discussion-Paragraphs 5 and 8)**

**Reviewer 2 - Results Comment 2:** Only GI changed among periodontal measurements, which limits the interpretation of periodontal parameter effects in the region. The effect of GI on FA should be explained, and relevant literature should be added.

**Response:** Thank you for this important comment. We clarified this point in the revised Discussion. Specifically, we now state more explicitly that because only the gingival index changed whereas PPD, PI, and BOP remained stable, the periodontal findings do not indicate a consistent local periodontal effect that could directly explain the observed increase in FD values. We further clarified that the isolated decrease in GI is more likely to reflect a general reduction in gingival inflammatory status during early healing rather than a direct determinant of peri-implant trabecular remodeling. In addition, we supported this interpretation with relevant literature and emphasized that clinical plaque/inflammation indices and radiographic fractal analysis provide complementary but distinct information. (**Discussion-Paragraph 5; References 19 and 26)**

**Reviewer 2 - Results Comment 3:** The lack of changes in MCW, ML, and PMI in the early period but changes in the long term could be discussed in more detail (why?).

**Response:** Thank you for this helpful comment. We expanded the Discussion to explain more clearly why MCW, ML, and PMI did not show significant changes during the early follow-up period. In the revised text, we now state that these indices are based on cortical and mental foramen-related morphometric measurements rather than on the local peri-implant trabecular ROI itself. Therefore, they may be less sensitive to short-term, site-specific trabecular remodeling during the early unloaded healing phase and may require longer follow-up to reflect more established structural adaptation. We also retained the comparison with previous studies reporting that cortical changes tend to become radiographically evident later than trabecular alterations. (**Discussion-Paragraph 2)**

**Reviewer 2 - Results Comment 4:** Advanced subgroup analyses are presented in the results; however, subgroups were not defined in the Materials and Methods section (implant placement regions, length, etc.).

**Response:** Thank you for this helpful comment. We revised the Materials and Methods section to define the subgroup analyses more clearly. In the revised manuscript, we now state that only posterior single-tooth implant sites were included and that subgroup analyses were performed according to jaw location (maxilla/mandible), implant length (8 mm/10 mm), and baseline periodontal status (gingivitis/periodontitis). These subgroup definitions are now explicitly described in the Methods section and correspond to the subgroup results presented in the revised Results/Table 4. **(Materials and Methods-Paragraphs 2 and 3; Results-Paragraph 5; Table 4)**

**Reviewer 2 - Results Comment 5:** Results for patients with gingivitis and periodontitis are presented, but these groups were not defined in the Materials and Methods section.

**Response:** Thank you for this helpful comment. We revised the Materials and Methods section to define the baseline periodontal status groups more clearly. In the revised manuscript, we now state explicitly that baseline periodontal status was classified as gingivitis or periodontitis according to the available clinical and radiographic records. This subgroup definition is now described in the Methods section and corresponds directly to the periodontal status comparisons presented in the revised subgroup table. **(Materials and Methods-Paragraph 3; Table 4)**

**Reviewer 2 - Results Comment 6:** Right-left comparisons are presented, but explanations are missing in the Materials and Methods section.

**Response:** Thank you for this helpful comment. We revised the Materials and Methods section to clarify the rationale for the right-left comparisons. In the revised manuscript, we now state that mandibular cortical width (MCW), mental length (ML), and panoramic mandibular index (PMI) were evaluated separately on the right and left sides because mandibular cortical and mental foramen-related measurements may show side-related variation on panoramic radiographs. This explanation has now been added explicitly to the Morphometric Measurements section. **(Materials and Methods-Paragraph 9)**

**Reviewer 2 - Results Comment 7:** Confidence interval information is missing.

**Response:** Thank you for this helpful comment. We revised the Results section to include confidence interval information for the primary outcome. Specifically, the change in mean peri-implant fractal dimension (FD) is now reported together with its 95% confidence interval (95% CI: 0.046 to 0.069) in the revised manuscript. **(Results-Paragraph 2)**

**Reviewer 2 - Discussion Comment 1:** A paragraph explaining and discussing the mechanism of periodontal parameter effects should be added. The current section is insufficient.

**Response:** Thank you for this important comment. We expanded the Discussion to clarify the interpretation of the periodontal findings in relation to the radiographic results. In the revised text, we now explain more explicitly that because only the gingival index changed whereas PPD, PI, and BOP remained stable, the periodontal findings do not indicate a consistent local periodontal effect that could directly explain the observed increase in FD values. We also clarified that the isolated decrease in GI is more likely to reflect a general reduction in gingival inflammatory status during early healing rather than a direct determinant of peri-implant trabecular remodeling. Accordingly, the periodontal variables and FA findings are now discussed as complementary but distinct dimensions of evaluation, while the main evidence of early structural bone change is derived from the radiographic FA findings. (**Discussion-Paragraph 5)**

**Reviewer 2 - Discussion Comment 2:** In line 232, the expressions standard care and routine panoramic are incorrectly used. Treatments are performed based on diagnosis, and radiographs are taken according to indications.

**Response:** Thank you for this helpful comment. We revised the relevant sentence in the Discussion accordingly. The expressions “routine panoramic radiographs” and “standard care” were removed, and the text now states more precisely that fractal analysis can be applied to panoramic radiographs obtained when clinically indicated during follow-up. This revision was made to align the wording more accurately with clinical decision-making and radiographic indication principles. (**Discussion-Paragraph 6)**

**Reviewer 2 - Discussion Comment 3:** The clinical significance of the study results should be strengthened. What is gained by detecting early changes?

**Response:** Thank you for this valuable comment. We revised the Discussion to strengthen the clinical significance of the findings more explicitly. In the revised text, we now clarify that the potential clinical value of detecting early FD changes lies in identifying trabecular reorganization during the healing period, even when conventional morphometric indices remain unchanged. We also state that this may support closer radiographic monitoring and more cautious interpretation of early healing patterns, particularly when additional mechanical stability assessment is not available. At the same time, we retained a cautious interpretation by emphasizing that FA should be regarded as a complementary radiographic tool rather than a standalone indicator of osseointegration or implant prognosis. (**Discussion-Paragraph 6)**

**Reviewer 2 - Discussion Comment 4:** Comparisons with the literature should be expanded.

**Response:** Thank you for this helpful comment. We expanded the literature comparisons throughout the revised Discussion. In particular, we added and strengthened comparisons with studies reporting postoperative increases in FD values and early trabecular reorganization, studies reporting discrepant or negative findings regarding FD change or prognostic utility, and recent literature emphasizing the methodological importance of ROI configuration in panoramic fractal analysis. We also incorporated additional comparisons regarding the clinical interpretation of FA findings and the influence of age and sex. These revisions were incorporated particularly in the revised Discussion. (**Discussion-Paragraphs 2,3,5 and 6; References 20,23,24 and 27)**

**Reviewer 2 - Conclusion Comment 1:** In line 269, in the phrase “Clear early alterations,” the word “clear” could be replaced with “detectable.”

**Response:** Thank you for this helpful suggestion. We revised the Conclusion accordingly and replaced the word “clear” with “detectable” to provide a more precise and appropriately cautious description of the early trabecular changes observed in the study. **(Conclusions)**

**Reviewer 2 - Tables Comment 1:** There are errors in emphasis in Tables 1–2–3 (normal/bold) (PI, PMI).

**Response:** Thank you for this helpful comment. We reviewed the formatting of the revised tables and corrected the emphasis of p-values so that only statistically significant comparisons are shown in bold. We also reorganized the tables in the revised manuscript, such that the primary FD results, the periodontal and morphometric comparisons, and the gender-based comparisons are now presented separately with consistent formatting and revised footnotes. The bold formatting of significant p-values has been checked accordingly in the revised tables. **(Tables 1-4)**

**Reviewer 2 - Tables Comment 2:** Tables 1–2–3 spelling error: panoramik → panoramic

**Response:** Thank you for this helpful comment. We reviewed the terminology used in the revised tables and corrected the spelling accordingly. The term “Panoramic Mandibular Index” is now used consistently and correctly throughout the revised tables and their footnotes. **(Tables 1-4)**

**Reviewer 2 - Tables Comment 3:** Table 1: FD (Fractal Dimension) is explained in the legend but not shown in the table.

**Response:** Thank you for this helpful comment. We agree that this was unclear in the previous version of the manuscript. In response, the tables were reorganized in the revised manuscript. The former Table 1, which contained periodontal and morphometric comparisons, is now presented as Table 2, and the primary fractal analysis results are now presented separately in the revised Table 1. Accordingly, FD (Fractal Dimension) is now explicitly shown in the revised Table 1 through the mesial FD, distal FD, apical FD, and mean peri-implant FD values, and the corresponding table footnotes were revised to match the table content consistently. **(Tables 1 and 2)**

**Reviewer 2 - Tables Comment 4:** Tables 1–2: P value → p should be lowercase.

**Response:** Thank you for this helpful comment. We revised the table formatting accordingly and standardized the notation to use lowercase p throughout the revised tables. In addition, following the reorganization of the table set, the former Tables 1 and 2 are now presented as Tables 2 and 3, respectively, and the corresponding column headings and footnotes were updated for consistency. **(Tables 2 and 3)**

**Reviewer 2 - Tables Comment 5:** Table 3 spelling error: Implant Localication → Localization

**Response:** Thank you for this helpful comment. We corrected this spelling error in the revised table by changing “Implant Localication” to “Localization.” In addition, following the reorganization of the table set, the former Table 3 is now presented as Table 4, where this subgroup heading appears in its corrected form. **(Table 4)**

**Reviewer 2 - Figures Comment 1:** Figure resolutions are very low. They are not usable.

**Response:** Thank you for this important comment. We revised the figures to improve both visual quality and readability. Higher-quality versions were prepared and replaced in the revised submission so that the anatomical reference points, measurement lines, and ROI annotations can be seen more clearly. The updated figure files have now been included in the revised submission. **(Figures 1 and 2; Figure Legends)**
