# Supplementary material for: Evaluation of early trabecular changes around implants using fractal analysis and panoramic indices
Source: PLoS One. 2026 Jul 1;21(7):e0352984. doi: 10.1371/journal.pone.0352984 (PMC13322550; doi:10.1371/journal.pone.0352984)
Supplement: Attachment — Submitted filename: Response_to_Reviewers_auresp_2.docx [file pone.0352984.s004.docx]

Dear Academic Editor and Reviewers,

We thank you for the careful evaluation of our manuscript entitled “Evaluation of early trabecular changes around implants using fractal analysis and panoramic indices.” We appreciate the constructive comments and have revised the manuscript accordingly. The major revisions include a more detailed and reproducible ROI standardization protocol, harmonization of terminology throughout the manuscript, clarification of the crestal bone loss-related exclusion criterion, improvement of figure quality and annotations, and provision of anonymized measurement data as S1 Data. Point-by-point responses are provided below.

**Response to Academic Editor and Reviewer #2**

**Comment 1:** ROI selection and reproducibility

The description of ROI selection is not sufficiently clear or reproducible. Although the manuscript states that “the mesial and distal ROIs were positioned parallel to the implant surface along the peri-implant trabecular bone” and that the apical ROI was placed immediately apical to the implant apex, the exact anatomical and radiographic reference points used for ROI placement should be described in greater detail.

Due to the two-dimensional nature of panoramic radiographs, magnification, distortion, and superimposition are inevitable, particularly in images obtained at different follow-up intervals. These factors may especially affect fractal dimension measurements performed on small ROIs. Since the main objective of the study is to evaluate changes in bone structure, even minor differences in ROI positioning may influence the results. Therefore, the authors should provide a clearer and more reproducible explanation of ROI standardization.

In addition, the rationale for the selected ROI size and the specific methodological details derived from references 15 and 16 should be explicitly explained rather than only cited.

**Response:** We thank the editor/reviewer for this important comment. We agree that ROI selection is a critical methodological step in fractal analysis performed on panoramic radiographs, because magnification, geometric distortion, and anatomical superimposition may influence FD measurements, particularly when small peri-implant ROIs are used. In response, we substantially revised the Fractal analysis procedure subsection of the Methods to provide a clearer and more reproducible description of ROI placement, standardization, baseline–follow-up matching, and ROI-size rationale.

Specifically, we clarified that baseline and 3-month panoramic radiographs were evaluated side by side to reproduce ROI placement at comparable peri-implant levels. Because baseline radiographs were obtained before implant placement, we further clarified that the implant long axis and the perpendicular apical reference line passing through the implant apex were first defined on the 3-month radiograph using the visible implant contour and apex. The corresponding baseline ROI locations were then reproduced on the preoperative radiograph by matching the implant recipient site with stable anatomical and radiographic landmarks, including adjacent root contours, alveolar crest morphology, edentulous ridge configuration, surrounding trabecular pattern, and region-specific anatomical boundaries such as the maxillary sinus floor, mandibular canal, or mental foramen when present. We also clarified that side-by-side assessment was used only to identify corresponding anatomical locations for ROI placement and not to visually judge changes in FD values.

We also added a geometric standardization protocol based on the implant long axis and the perpendicular apical reference line. ROI-1, ROI-2, and ROI-3 were defined as the mesial, distal, and apical peri-implant trabecular ROIs, respectively. ROI-1 and ROI-2 were oriented parallel to the implant long axis, with their inferior borders aligned with the apical reference line, and were positioned in the closest available mesial and distal peri-implant trabecular bone adjacent to the implant surface without including the implant threads or implant surface. ROI-3 was positioned immediately apical to the implant apex, with its superior border aligned with the same apical reference line and centered on the implant long axis.

We further clarified the exclusion boundaries during ROI placement. The implant surface, implant threads, cortical bone, lamina dura, adjacent roots, sinus floor, mandibular canal, radiopaque restorative materials, and areas of anatomical superimposition or image artifact were excluded from the ROIs. We also added that when the predefined ROI could not be reproducibly placed within trabecular bone without including these structures, that region was not measured. This rule was applied to both baseline and follow-up images to reduce measurement variability related to panoramic distortion and superimposition.

The rationale for the selected ROI dimensions was also expanded. The 25 × 50 pixel mesial and distal ROIs were selected to sample the narrow peri-implant trabecular zone along the implant surface while minimizing inclusion of the implant margin, adjacent root structures, or cortical borders. The 50 × 50 pixel apical ROI was selected to sample the trabecular area immediately apical to the implant apex, where a broader trabecular compartment was usually available. These ROI dimensions were kept fixed across patients and time points to obtain comparable measurements and to provide sufficient trabecular information for box-counting analysis. We also clarified that this approach was selected with reference to previous dental fractal analysis studies using fixed ROI dimensions and to evidence indicating that ROI size, configuration, and placement may influence FD measurements.

In addition, Fig 1 and its legend were revised to more clearly illustrate and define the standardized ROI placement. The revised Fig 1B now provides an enlarged visual representation of the peri-implant ROI placement protocol, including the implant long axis and the perpendicular apical reference line passing through the implant apex. The figure legend was also updated to explain that the corresponding baseline ROI locations were reproduced by side-by-side matching of stable anatomical landmarks, and to identify the location of ROI-1, ROI-2, and ROI-3 in relation to these reference lines.

Finally, we added a statement to the limitations section acknowledging that, despite the use of fixed ROI dimensions and reproducible radiographic landmarks, minor variations in ROI positioning cannot be completely excluded because of the inherent magnification, geometric distortion, and anatomical superimposition associated with panoramic radiography. This limitation is now explicitly stated to guide cautious interpretation of subtle FD changes. **(Materials and methods:** **Fractal analysis procedure; Figure Legends: Figure 1; Discussion: Paragraph 7)**

**Comment 2:** Terminology should be used consistently throughout the manuscript. The terms “early structural changes,” “early healing,” and “trabecular remodelling” appear to be used interchangeably. The authors should select a single main conceptual framework and use it consistently throughout the title, abstract, introduction, discussion, and conclusion.

**Response:** We thank the editor/reviewer for this important comment. We agree that the terminology should be used consistently throughout the manuscript and that the terms “early structural changes,” “early healing,” and “trabecular remodelling” should not be used interchangeably.

We retained the title because it already uses the neutral term “early trabecular changes,” which is consistent with the radiographic nature of the study and does not imply direct evidence of osseointegration or histological remodeling. However, in response to the comment, we revised the abstract, introduction, results, discussion, and conclusion to harmonize the terminology throughout the manuscript.

The main conceptual framework of the revised manuscript is now early trabecular changes, more specifically early peri-implant trabecular structural changes where appropriate. The term “early healing” is now used only to describe the clinical time interval, namely the 3-month unloaded healing period, rather than as the primary radiographic outcome. Similarly, terms such as “trabecular remodelling” were replaced where appropriate with more neutral terms such as “trabecular structural changes” or “radiographic trabecular structural changes.”

We also revised interpretive statements to avoid overinterpretation of the fractal analysis findings. In particular, we clarified that fractal analysis detects radiographic changes in trabecular microarchitecture and should not be interpreted as direct evidence of osseointegration or histological remodeling. These revisions were made to improve conceptual consistency and to ensure that the terminology accurately reflects the radiographic and exploratory nature of the study findings. **(Abstract; Introduction: Paragraphs 4 and 5; Results: Paragraph 2; Discussion: Paragraphs 1-5 and 6-7; Conclusions)**

**Comment 3:** Language and punctuation. The language, grammar, and punctuation of the manuscript should be carefully reviewed. Several sentences would benefit from editing to improve clarity, academic flow, and readability.

**Response:** We thank the editor/reviewer for this comment. We have carefully revised the manuscript for language, grammar, punctuation, clarity, and academic flow. Long or potentially ambiguous sentences were edited, and several sections were rephrased to improve readability, particularly the Abstract, Methods, Discussion, and Conclusion.

As part of this revision, we also harmonized terminology throughout the manuscript and revised interpretive statements to avoid overstatement of the radiographic findings. In addition, minor punctuation, spacing, abbreviation, and formatting errors were corrected throughout the text.

We believe that these revisions have improved the overall clarity, consistency, and readability of the manuscript.

**Comment 4:** Exclusion criterion related to crestal bone loss. The exclusion criterion “findings of crestal bone loss in follow-up images” should be reconsidered. Excluding cases with crestal bone loss may introduce selection bias and may shift the results in a more favorable direction. Since crestal bone loss is clinically relevant to peri-implant bone changes, the authors should either provide a clear justification for this exclusion criterion or consider including such cases with appropriate subgroup or sensitivity analysis.

**Response:** We thank the editor/reviewer for this important comment. We agree that excluding cases solely because crestal bone loss is present on follow-up radiographs could introduce selection bias and may shift the results in a more favorable direction. In response, we revised this exclusion criterion to clarify that crestal bone loss was not used as an exclusion criterion by itself.

In the revised manuscript, this exclusion criterion was reworded to indicate that radiographs were excluded only when crestal bone loss, peri-implant radiolucency, cortical disruption, or related radiographic changes prevented reproducible placement of the predefined trabecular ROIs. We also clarified that cases were not excluded solely on the basis of clinically detectable crestal bone loss; exclusion was applied only when radiographic changes prevented standardized ROI placement without including cortical defects, radiolucent areas, the implant surface, or anatomical superimposition.

This clarification was made to distinguish a technical radiographic exclusion criterion related to ROI reproducibility from a clinical exclusion criterion based on peri-implant bone status. Because the primary outcome of the study was based on FD measurements obtained from predefined trabecular ROIs, reliable and reproducible ROI placement was necessary to reduce measurement variability related to panoramic distortion, anatomical superimposition, and altered peri-implant anatomy.

In addition, we added a statement to the limitations section acknowledging that, although this approach may have reduced measurement variability, it may also limit the generalizability of the findings to implants with radiographically stable peri-implant bone conditions. **(Materials and methods: Paragraphs 5 and 6; Discussion: Paragraph 7)**

**Comment 5:** Figure quality and resolution. The resolution and quality of the figures should be improved. The current figures are not sufficiently clear for evaluating ROI placement, measurement points, and radiographic details. Higher-resolution images should be provided, and all annotations, labels, and arrows should be made clearly visible. This is particularly important for figures demonstrating ROI selection and fractal analysis procedures.

**Response:** We thank the editor/reviewer for this comment. We agree that high-quality figures are essential for evaluating ROI placement, measurement points, radiographic landmarks, and the fractal analysis workflow. In response, we revised the figure files and improved the visibility of annotations, labels, arrows, ROI boundaries, reference lines, and measurement lines.

Fig 1 was revised into two panels to improve readability at publication size. Fig 1A now shows the panoramic morphometric measurements, including MCW, ML, PMI, and the mental foramen reference. Fig 1B provides an enlarged view of the peri-implant ROI placement. In this enlarged panel, the ROI boundaries, reference lines, arrows, and labels were made more distinct to improve visualization of ROI-1, ROI-2, and ROI-3. The figure legend was revised accordingly to define the blue longitudinal line as the implant long axis and the blue transverse line as the perpendicular apical reference line, and to clarify the standardized positions of the ROIs.

Fig 2 was also reviewed and prepared in higher resolution to improve the visibility of the fractal analysis workflow, panel labels, processing steps, and box-counting output. Revised high-resolution TIFF files have been uploaded with the revised manuscript. **(Figures 1 and 2; Figure Legends)**

**Reviewer #1 Comment:** All comments have been addressed

**Response:** We thank Reviewer #1 for the positive assessment and for indicating that all previous comments have been addressed.

**Additional comment. Data availability**

**Reviewer comment:** Has the author made all data underlying the findings in their manuscript fully available?

**Reviewer #1:** Yes **Reviewer #2:** No

**Response:** We thank the reviewer for this comment. We acknowledge the importance of making all data underlying the findings available in accordance with the PLOS ONE data policy. In the revised submission, we have provided the anonymized measurement dataset underlying the findings as S1 Data.

The S1 Data file includes the de-identified clinical and radiographic measurement data used in the analyses, including FD values, panoramic morphometric measurements, periodontal parameters, and relevant grouping variables. The dataset therefore provides the data points underlying the reported means, standard deviations, and statistical comparisons.

To protect participant confidentiality, all potentially identifying information, including patient names, institutional record numbers, radiographic identifiers, exact imaging dates, and other direct or indirect identifiers, has been removed from the dataset. Raw panoramic radiographs are not publicly available because they contain potentially identifying patient information and were obtained from retrospective clinical archives under ethics committee approval.

Accordingly, the Data Availability Statement was revised to clarify that all anonymized measurement data underlying the findings are provided as Supporting Information, whereas raw radiographic images are not publicly available due to participant privacy considerations. **(Data availability; S1 Data)**
