# Supplementary material for: Evaluation of early trabecular changes around implants using fractal analysis and panoramic indices
Source: PLoS One. 2026 Jul 1;21(7):e0352984. doi: 10.1371/journal.pone.0352984 (PMC13322550; doi:10.1371/journal.pone.0352984)
Supplement: Peer review history

# Peer review: Decision Letter 0

**DOI:** 10.1371/journal.pone.0352984.r001

**Authors:** Katibe Tugce Temur

13 Apr 2026

PONE-D-26-11186

Evaluation of early trabecular changes around implants using fractal analysis and panoramic indices

PLOS One

Dear Dr. keles,

Thank you for submitting your manuscript to PLOS ONE. After careful consideration, we feel that it has merit but does not fully meet PLOS ONE’s publication criteria as it currently stands. Therefore, we invite you to submit a revised version of the manuscript that addresses the points raised during the review process.

==============================

**ACADEMIC EDITOR:**

Dear Keles,

The manuscript addresses a relevant research question; however, based on both the reviewers’ comments and my evaluation, major revisions are required before the manuscript can be considered for publication in PLOS ONE.

The study rationale should be clarified, particularly the role and contribution of clinical periodontal parameters in relation to peri-implant bone healing.

The inconsistency in ethics approval information must be corrected, and all related details (approval number, committee name, and date) should be fully consistent throughout the manuscript and submission system.

The Materials and Methods section requires substantial clarification, including power analysis, participant characteristics (e.g., age range), ROI selection and reproducibility, and clear definitions of all subgroups (implant region, implant length, periodontal status, etc.).

The presentation of results should be improved. Tables and figures must be revised for clarity, consistency, and accuracy. In particular, Table 3 should be restructured, and figure quality should be significantly improved.

The discussion should be revised to provide a more cautious interpretation of the findings, considering the retrospective design, sample size, and follow-up period.

Language and terminology should be carefully revised to correct spelling errors and ensure consistency throughout the manuscript.

The introduction could be improved by presenting the originality and clinical relevance of fractal analysis in a more balanced manner.

Additional recent literature may be incorporated to support the interpretation of findings.

Visual presentation of morphometric measurements (e.g., mandibular indices) could be enhanced for better reader understanding.

==============================

Please submit your revised manuscript by May 28 2026 11:59PM. If you will need more time than this to complete your revisions, please reply to this message or contact the journal office at plosone@plos.org. When you're ready to submit your revision, log on to https://www.editorialmanager.com/pone/ and select the 'Submissions Needing Revision' folder to locate your manuscript file.

Please include the following items when submitting your revised manuscript:

- A letter that responds to each point raised by the academic editor and reviewer(s). You should upload this letter as a separate file labeled 'Response to Reviewers'.
- A marked-up copy of your manuscript that highlights changes made to the original version. You should upload this as a separate file labeled 'Revised Manuscript with Track Changes'.
- An unmarked version of your revised paper without tracked changes. You should upload this as a separate file labeled 'Manuscript'.

If you would like to make changes to your financial disclosure, please include your updated statement in your cover letter. Guidelines for resubmitting your figure files are available below the reviewer comments at the end of this letter.

If applicable, we recommend that you deposit your laboratory protocols in protocols.io to enhance the reproducibility of your results. Protocols.io assigns your protocol its own identifier (DOI) so that it can be cited independently in the future. For instructions see: https://journals.plos.org/plosone/s/submission-guidelines#loc-laboratory-protocols. Additionally, PLOS ONE offers an option for publishing peer-reviewed Lab Protocol articles, which describe protocols hosted on protocols.io. Read more information on sharing protocols at https://plos.org/protocols?utm_medium=editorial-email&utm_source=authorletters&utm_campaign=protocols.

As the corresponding author, your ORCID iD is verified in the submission system and will appear in the published article. PLOS supports the use of ORCID, and we encourage all coauthors to register for an ORCID iD and use it as well. Please encourage your coauthors to verify their ORCID iD within the submission system before final acceptance, as unverified ORCID iDs will not appear in the published article. *Only*  the individual author can complete the verification step; PLOS staff *cannot*  verify ORCID iDs on behalf of authors.

We look forward to receiving your revised manuscript.

Kind regards,

Dr. Katibe Tugce Temur

Academic Editor

PLOS One

Journal Requirements:

When submitting your revision, we need you to address these additional requirements.

1. Please ensure that your manuscript meets PLOS ONE's style requirements, including those for file naming. The PLOS ONE style templates can be found at

https://journals.plos.org/plosone/s/file?id=wjVg/PLOSOne_formatting_sample_main_body.pdf and

https://journals.plos.org/plosone/s/file?id=ba62/PLOSOne_formatting_sample_title_authors_affiliations.pdf

2. The ethical approval number(s) listed in the manuscript and/or submission metadata does not match the approval number on the ethical approval document you provided. Please ensure that all approval numbers are correct

3. We note that there is identifying data in the Supporting Information file S1 File.xlsx. Due to the inclusion of these potentially identifying data, we have removed this file from your file inventory. Prior to sharing human research participant data, authors should consult with an ethics committee to ensure data are shared in accordance with participant consent and all applicable local laws.

Data sharing should never compromise participant privacy. It is therefore not appropriate to publicly share personally identifiable data on human research participants. The following are examples of data that should not be shared:

-Name, initials, physical address

-Ages more specific than whole numbers

-Internet protocol (IP) address

-Specific dates (birth dates, death dates, examination dates, etc.)

-Contact information such as phone number or email address

-Location data

-ID numbers that seem specific (long numbers, include initials, titled “Hospital ID”) rather than random (small numbers in numerical order)

Data that are not directly identifying may also be inappropriate to share, as in combination they can become identifying. For example, data collected from a small group of participants, vulnerable populations, or private groups should not be shared if they involve indirect identifiers (such as sex, ethnicity, location, etc.) that may risk the identification of study participants.

Additional guidance on preparing raw data for publication can be found in our Data Policy (https://journals.plos.org/plosone/s/data-availability#loc-human-research-participant-data-and-other-sensitive-data) and in the following article: http://www.bmj.com/content/340/bmj.c181.long.

Please remove or anonymize all personal information age, ensure that the data shared are in accordance with participant consent, and re-upload a fully anonymized data set. Please note that spreadsheet columns with personal information must be removed and not hidden as all hidden columns will appear in the published file.

4. Your ethics statement should only appear in the Methods section of your manuscript. If your ethics statement is written in any section besides the Methods, please move it to the Methods section and delete it from any other section. Please ensure that your ethics statement is included in your manuscript, as the ethics statement entered into the online submission form will not be published alongside your manuscript.

5. Please include captions for your Supporting Information files at the end of your manuscript, and update any in-text citations to match accordingly. Please see our Supporting Information guidelines for more information: http://journals.plos.org/plosone/s/supporting-information.

6. If the reviewer comments include a recommendation to cite specific previously published works, please review and evaluate these publications to determine whether they are relevant and should be cited. There is no requirement to cite these works unless the editor has indicated otherwise.

Additional Editor Comments:

Dear Authors,

The manuscript addresses a clinically relevant topic; however, based on the reviewers’ comments, substantial revisions are required before it can be considered for publication.

Several important issues need to be addressed. First, the rationale of the study should be clarified, particularly regarding the inclusion of clinical periodontal parameters alongside peri-implant bone healing. Statements related to the predictive value of fractal analysis and the originality of the study should be presented in a more cautious and balanced manner.

There are also methodological and reporting inconsistencies that must be corrected. These include discrepancies in the ethics approval information, lack of power analysis, insufficient description of ROI selection and reproducibility, unclear definition of subgroups (implant region, length, periodontal status), and missing details such as age range and confidence intervals. The contribution of periodontal parameters to the analysis should be more clearly justified.

In addition, data presentation requires improvement. Tables and figures are not fully consistent with their descriptions, and some are difficult to interpret. Table structures, titles, and footnotes should be revised for clarity, and figure quality must be improved. Anatomical reference points and morphometric measurements should be more clearly illustrated.

The discussion section should be strengthened, particularly by adopting a more cautious interpretation of findings in light of the retrospective design, limited sample size, and follow-up period. Mechanistic explanations and comparisons with recent literature should be expanded.

Finally, language and formatting issues (e.g., spelling errors, inconsistent terminology, punctuation) should be carefully revised throughout the manuscript.

[Note: HTML markup is below. Please do not edit.]

Reviewers' comments:

Reviewer's Responses to Questions

**Comments to the Author**

1. Is the manuscript technically sound, and do the data support the conclusions?

The manuscript must describe a technically sound piece of scientific research with data that supports the conclusions. Experiments must have been conducted rigorously, with appropriate controls, replication, and sample sizes. The conclusions must be drawn appropriately based on the data presented.

Reviewer #1: Partly

Reviewer #2: Yes

**********

2. Has the statistical analysis been performed appropriately and rigorously?

Reviewer #1: Yes

Reviewer #2: I Don't Know

**********

3. Have the authors made all data underlying the findings in their manuscript fully available?

The PLOS Data policy requires authors to make all data underlying the findings described in their manuscript fully available without restriction, with rare exception (please refer to the Data Availability Statement in the manuscript PDF file). The data should be provided as part of the manuscript or its supporting information, or deposited to a public repository. For example, in addition to summary statistics, the data points behind means, medians and variance measures should be available. If there are restrictions on publicly sharing data—e.g. participant privacy or use of data from a third party—those must be specified.

Reviewer #1: Yes

Reviewer #2: Yes

**********

4. Is the manuscript presented in an intelligible fashion and written in standard English?

PLOS ONE does not copyedit accepted manuscripts, so the language in submitted articles must be clear, correct, and unambiguous. Any typographical or grammatical errors should be corrected at revision, so please note any specific errors here.

Reviewer #1: Yes

Reviewer #2: No

**********

5. Review Comments to the Author

Please use the space provided to explain your answers to the questions above. You may also include additional comments for the author, including concerns about dual publication, research ethics, or publication ethics. (Please upload your review as an attachment if it exceeds 20,000 characters)

Reviewer #1: The manuscript addresses a current and clinically relevant topic; however, some revisions are needed to improve the clarity and overall quality of the study.

Comment 1- In the introduction, although the main focus of the study is peri-implant bone healing, the rationale for including clinical periodontal parameters should be explained more clearly. In addition, if the statements regarding the value of fractal analysis in predicting implant success and the originality of the study are presented in a more cautious manner, the text will appear more balanced.

Comment 2- There is an inconsistency in the ethics approval information. In the ethics statement entered in the submission system, the ethics committee decision is reported as “2025/639” with the date “July 31, 2025,” whereas in the manuscript text it is stated as “2024/460.” This discrepancy should be corrected. The ethics approval number, committee name, and date should be fully consistent across all sections of the submission.

Comment 3- In the Materials and Methods section, the contribution of the periodontal parameters obtained from natural teeth to the study should be stated more clearly. Figure 1 could be improved by presenting the mandibular indices more clearly. A more explicit visualization of the anatomical reference points and measured parameters would help the reader better understand the morphometric assessment.

Comment 4- In Table 3, implant location and implant length are presented together; however, the table title and footnote are not fully consistent with the actual structure of the table. In addition, the footnote refers to “implant design” and “implant region,” although these variables are not clearly presented in the table itself. In its current form, the table is difficult to read and somewhat confusing. A clearer presentation would be preferable, ideally by separating these results into different tables and indicating more explicitly which comparison each p-value refers to.

Comment 5- In the discussion section, expressing the comments regarding the clinical utility of fractal analysis and the limited influence of age/gender in a more cautious manner, taking into account the retrospective design, short follow-up period, and sample size of the study, could strengthen the discussion. It may also be useful to include recent literature supporting the use of fractal analysis on panoramic radiographs for the assessment of trabecular bone microarchitecture (e.g., doi: 10.1186/s12880-026-02193-7).

Comment 6- In the conclusion section, it may be helpful to express the comparison with clinical parameters more cautiously, since these measurements were obtained not from the implant sites but from natural teeth in the same quadrant.

Comment 7- Some language and spelling issues should also be corrected. For example, the term “fractal dimesion” is misspelled in the abstract. Similarly, the expressions “Panoramic Mandibular Index” and “Panoramic Mental Index” are not used consistently across the tables. Although the manuscript is generally understandable, it would benefit from careful language editing in terms of terminology and formal presentation.

Reviewer #2: Abstract

1. There are spelling errors in the abstract section. It should be reviewed again. For example, (fractal dimesion → dimension).

Introduction

1. "have gained widespread acceptance"

→ can be revised as "are widely accepted."

2. The reason for preferring 3 months in the early-period evaluation should be explained in more detail.

3. An example of an early-period study should be added, and the contribution of the present study to early-period research should be emphasized.

4. The effect of periodontal parameters in the hypothesis remains unclear. Further clarification is required.

5. In line 66, the expression radiographs.1 is incorrect.

6. In line 69, an em dash (—) should be used:

‘for implant success—particularly in the early healing phase—requires further investigation.’

Materials and Methods

1. The ethics approval numbers do not match those in the Ethics Statement section.

2. Power analysis is missing.

3. The age range of participants has not been specified.

4. The reproducibility of ROI selection is unclear. Reference points used for selecting mesial-distal and apical regions could be added.

5. The rationale for ROI size should be explained.

6. Implant placement regions (anterior/posterior) should be included. The region may influence analysis results.

7. The use of ‘-’ vs. ‘—’ (em dash) and commas should be reviewed.

Results

1. The relationship between periodontal measurements and the region of interest is weak. The relationship between periodontal parameters and FA should be explained.

2. Only GI changed among periodontal measurements, which limits the interpretation of periodontal parameter effects in the region. The effect of GI on FA should be explained, and relevant literature should be added.

3. The lack of changes in MCW, ML, and PMI in the early period but changes in the long term could be discussed in more detail (why?).

4. Advanced subgroup analyses are presented in the results; however, subgroups were not defined in the Materials and Methods section (implant placement regions, length, etc.).

5. Results for patients with gingivitis and periodontitis are presented, but these groups were not defined in the Materials and Methods section.

6. Right-left comparisons are presented, but explanations are missing in the Materials and Methods section.

7. Confidence interval information is missing.

Discussion

1. A paragraph explaining and discussing the mechanism of periodontal parameter effects should be added. The current section is insufficient.

2. In line 232, the expressions standard care and routine panoramic are incorrectly used. Treatments are performed based on diagnosis, and radiographs are taken according to indications.

3. The clinical significance of the study results should be strengthened. What is gained by detecting early changes?

4. Comparisons with the literature should be expanded.

Conclusion

1. In line 269, in the phrase “Clear early alterations,” the word “clear” could be replaced with “detectable.”

Tables

1. There are errors in emphasis in Tables 1–2–3 (normal/bold) (PI, PMI).

2. Tables 1–2–3 spelling error: panoramik → panoramic

3. Table 1: FD (Fractal Dimension) is explained in the legend but not shown in the table.

4. Tables 1–2: P value → p should be lowercase.

5. Table 3 spelling error: Implant Localication → Localization

Figures

1. Figure resolutions are very low. They are not usable.

**********

6. PLOS authors have the option to publish the peer review history of their article (what does this mean?). If published, this will include your full peer review and any attached files.

If you choose “no”, your identity will remain anonymous but your review may still be made public.

**Do you want your identity to be public for this peer review?**  For information about this choice, including consent withdrawal, please see our Privacy Policy.

Reviewer #1: No

Reviewer #2: No

**********

[NOTE: If reviewer comments were submitted as an attachment file, they will be attached to this email and accessible via the submission site. Please log into your account, locate the manuscript record, and check for the action link "View Attachments". If this link does not appear, there are no attachment files.]

To ensure your figures meet our technical requirements, please review our figure guidelines: https://journals.plos.org/plosone/s/figures

You may also use PLOS’s free figure tool, NAAS, to help you prepare publication quality figures: https://journals.plos.org/plosone/s/figures#loc-tools-for-figure-preparation.

NAAS will assess whether your figures meet our technical requirements by comparing each figure against our figure specifications.

# Author comment: Author response to Decision Letter 1

**DOI:** 10.1371/journal.pone.0352984.r002

28 Apr 2026

Response to Academic Editor

Academic Editor - Comment 1: The study rationale should be clarified, particularly the role and contribution of clinical periodontal parameters in relation to peri-implant bone healing.

Response: Thank you for this important comment. We have revised the manuscript to clarify the study rationale and to better define the role of the clinical periodontal parameters. In the revised Introduction, we emphasized that the primary aim of the study was to evaluate early peri-implant alveolar bone changes by comparing fractal dimension (FD) values obtained from panoramic radiographs at baseline and at 3 months postoperatively. We also clarified that the analyses involving panoramic morphometric indices and clinical periodontal parameters were secondary and exploratory in nature. In the Materials and Methods section, we specified that the periodontal parameters were obtained from natural teeth in the same quadrant and were evaluated as secondary clinical variables for exploratory comparison with the radiographic findings. In addition, the Discussion and Conclusion sections were revised to present these periodontal findings more cautiously, as supportive clinical context rather than direct indicators of peri-implant bone healing. These revisions were made to ensure a clearer distinction between the primary radiographic objective of the study and the supplementary role of the clinical periodontal parameters. (Introduction-Paragraphs 1,3,4 and 5; Materials and Methods-Paragraph 3; Results-Paragraphs 2,3 and 5; Discussion-Paragraphs 3,4,5 and 6; Conclusions)

Academic Editor - Comment 2: The inconsistency in ethics approval information must be corrected, and all related details (approval number, committee name, and date) should be fully consistent throughout the manuscript and submission system.

Response: Thank you for pointing this out. We carefully reviewed the ethics approval document and corrected the manuscript accordingly. The correct ethics approval information is now reported consistently as Necmettin Erbakan University Faculty of Dentistry Non-Drug and Non-Medical Device Research Ethics Committee, Approval No. 2025/639, dated 31.07.2025. We also revised the submission metadata to ensure that the committee’s name, approval number, and date are fully consistent across all parts of the submission. In addition, in accordance with the journal’s requirement, the ethics statement was retained only in the Materials and Methods section and removed from the separate Ethics statement section. (Materials and Methods-Paragraph 1)

Academic Editor - Comment 3: The Materials and Methods section requires substantial clarification, including power analysis, participant characteristics (e.g., age range), ROI selection and reproducibility, and clear definitions of all subgroups (implant region, implant length, periodontal status, etc.).

Response: Thank you for this helpful comment. We substantially revised the Materials and Methods section to improve methodological clarity and transparency. Specifically, we added the age range of the study population and clarified the participant characteristics. We also defined the primary outcome variable as the change in mean peri-implant fractal dimension (FD), calculated as the average of the mesial and distal FD values between baseline and 3 months, and included a post hoc power analysis based on this outcome. In addition, we expanded the description of ROI selection by specifying the positioning of the mesial, distal, and apical ROIs, the reference points used for standardization, and the rationale for the selected ROI dimensions. We further clarified the subgroup definitions used in the analyses, including implant localization, implant length, and baseline periodontal status, and we specified that morphometric measurements were evaluated separately on the right and left sides because of possible side-related variation on panoramic radiographs. To improve consistency between the Methods, Results, and data presentation, we also revised and reorganized the tables so that the primary FD analysis, secondary periodontal and morphometric analyses, gender-based comparisons, and exploratory subgroup analyses are now presented more clearly and in alignment with the revised analytical framework. (Materials and Methods-Paragraphs 2,3,8,9 and 10; Results-Paragraph 2; Tables 1-4)

Academic Editor - Comment 4: The presentation of results should be improved. Tables and figures must be revised for clarity, consistency, and accuracy. In particular, Table 3 should be restructured, and figure quality should be significantly improved.

Response: Thank you for this helpful comment. We revised the presentation of the Results section to improve clarity, consistency, and accuracy. The tables were reorganized to better reflect the analytical structure of the study. Specifically, the primary FD analysis, the secondary periodontal and morphometric comparisons, the gender-based comparisons, and the exploratory subgroup analyses are now presented in separate tables with revised titles, footnotes, and table numbering. The former subgroup table was restructured to improve readability and to ensure full consistency between its content, heading, and explanatory note. We also reviewed the tables for numerical consistency with the revised dataset and updated the Results text accordingly. In addition, the figures were revised to improve visual clarity and to better illustrate the anatomical reference points and measured parameters. (Results-Paragraphs 2-5; Tables 1-4; Figure 1)

Academic Editor - Comment 5: The discussion should be revised to provide a more cautious interpretation of the findings, considering the retrospective design, sample size, and follow-up period.

Response: Thank you for this important comment. We revised the Discussion and Conclusion sections to provide a more cautious interpretation of the findings. Specifically, we tempered the clinical interpretation of the observed FD changes and avoided overstatement regarding the role of fractal analysis in implant healing. We emphasized that the study was retrospective in design, included a modest sample size, and was limited to a 3-month follow-up period, which precludes definitive conclusions regarding long-term bone remodeling or osseointegration. We also clarified that fractal analysis should be interpreted as a complementary radiographic method reflecting early trabecular reorganization rather than as a standalone or confirmatory indicator of osseointegration. In addition, the limitations related to the study design, follow-up duration, and the contextual nature of the periodontal variables were made more explicit in the revised Discussion. The Conclusion section was also revised accordingly to maintain a more cautious and balanced interpretation. (Results-Paragraphs 2 and 5; Discussion-Paragraphs 3,5,6 and 7; Conclusions)

Academic Editor - Comment 6: Language and terminology should be carefully revised to correct spelling errors and ensure consistency throughout the manuscript.

Response: Thank you for this helpful comment. We carefully revised the manuscript for language, spelling, punctuation, and terminology consistency throughout the text, tables, and figure legends. Several typographical errors were corrected, and terminology was standardized across all sections of the manuscript. In particular, radiographic and morphometric terms, subgroup labels, and abbreviations were reviewed to ensure consistent usage in the main text, tables, and explanatory notes. We also corrected formatting and wording inconsistencies that arose during revision to improve the overall clarity and readability of the manuscript.

Academic Editor - Comment 7: The introduction could be improved by presenting the originality and clinical relevance of fractal analysis in a more balanced manner.

Response: Thank you for this valuable comment. We revised the Introduction to present the originality and clinical relevance of fractal analysis in a more balanced and cautious manner. Specifically, we reduced overly strong wording regarding the potential role of fractal analysis, clarified that its value in detecting early peri-implant trabecular changes on panoramic radiographs remains an area requiring further investigation, and emphasized the study’s focus on early radiographic assessment rather than definitive prediction of implant success. We also restructured the rationale of the study to better distinguish the primary radiographic objective from the secondary exploratory analyses. (Introduction-Paragraphs 1-5)

Academic Editor - Comment 8: Additional recent literature may be incorporated to support the interpretation of findings.

Response: Thank you for this helpful suggestion. We incorporated additional recent literature into the revised manuscript to strengthen and update the interpretation of our findings. The newly added references address peri-implant bone architecture, early trabecular remodeling, the interpretive limits of fractal analysis in implant-related outcomes, and the methodological importance of ROI configuration in panoramic radiographic assessment. These references were integrated into the Discussion section to provide a more current and balanced framework for interpreting the observed FD changes and to improve comparison with recent evidence. (Discussion-Paragraphs 4 and 6; References 20,23 and 24)

Academic Editor - Comment 9: Visual presentation of morphometric measurements (e.g., mandibular indices) could be enhanced for better reader understanding.

Response: Thank you for this helpful suggestion. We revised the visual presentation of the morphometric measurements to improve reader understanding. Figure 1 was updated to show the MCW measurement, the mental foramen reference point, and the peri-implant ROI locations more clearly, with improved image quality and clearer annotations. The figure legend was also revised accordingly to provide a more explicit explanation of the anatomical landmarks and measured parameters. (Figure 1; Figure Legends)

Journal Requirements

Comment 1: Please ensure that your manuscript meets PLOS ONE's style requirements, including those for file naming.

Response: Thank you for this helpful comment. We carefully revised the manuscript to align it more closely with PLOS ONE style requirements. Specifically, we updated the title and affiliation formatting, standardized section headings, placed figure captions and tables after their first citation in the text, and revised the supporting information section accordingly. We also checked the file naming and overall submission formatting to improve consistency with PLOS ONE requirements.

Comment 2: The ethical approval number(s) listed in the manuscript and/or submission metadata does not match the approval number on the ethical approval document you provided. Please ensure that all approval numbers are correct.

Response: Thank you for pointing this out. We carefully reviewed the ethics approval document and corrected the manuscript and submission metadata accordingly. The correct ethics approval information is now reported consistently throughout the submission as Necmettin Erbakan University Faculty of Dentistry Non-Drug and Non-Medical Device Research Ethics Committee, Approval No. 2025/639, dated 31.07.2025. We also checked the relevant sections of the manuscript to ensure full consistency in the committee name, approval number, and date. (Materials and Methods-Paragraph 1)

Comment 3: We note that there is identifying data in the Supporting Information file S1 File.xlsx. Due to the inclusion of these potentially identifying data, we have removed this file from your file inventory. Prior to sharing human research participant data, authors should consult with an ethics committee to ensure data are shared in accordance with participant consent and all applicable local laws. Please remove or anonymize all personal information, ensure that the data shared are in accordance with participant consent, and re-upload a fully anonymized data set. Please note that spreadsheet columns with personal information must be removed and not hidden, as all hidden columns will appear in the published file.

Response: Thank you for this important note. We re-examined the original supporting spreadsheet and prepared a fully anonymized replacement file. All potentially identifying information was removed from the dataset, and no identifying columns were retained in hidden form. The revised supporting file now includes only de-identified measurement data necessary to support the reported findings. We also confirmed that the shared dataset is consistent with participant privacy protection and the retrospective use of anonymized data. The corrected file has been re-uploaded as S1 Data. (Supporting information-S1 Data)

Comment 4: Your ethics statement should only appear in the Methods section of your manuscript. If your ethics statement is written in any section besides the Methods, please move it to the Methods section and delete it from any other section. Please ensure that your ethics statement is included in your manuscript, as the ethics statement entered into the online submission form will not be published alongside your manuscript.

Response: Thank you for this clarification. We revised the manuscript accordingly so that the ethics statement now appears only in the Materials and Methods section. Any duplicate ethics statement presented elsewhere in the manuscript was removed. We also confirmed that the full ethics approval information remains included in the manuscript text, in accordance with the journal’s requirements. (Materials and Methods-Paragraph 1)

Comment 5: Please include your Supporting Information captions at the end of the manuscript file. If any Supporting Information files are cited in the manuscript, please ensure that the file names and captions are presented consistently.

Response: Thank you for this clarification. We revised the manuscript accordingly and added the Supporting Information caption at the end of the manuscript file. We also reviewed the manuscript for consistency between the in-text citation, file label, and uploaded supporting file. The supporting dataset is now cited and labeled consistently as S1 Data throughout the revised submission. (S1 Data; Supporting information)

Comment 6: Please consider the reviewer and editor suggestions regarding additional relevant literature and revise the manuscript accordingly.

Response: Thank you for this note. We carefully reviewed the publications recommended in the reviewer comments and evaluated their relevance to the scope, methodology, and interpretation of the present study. Relevant works were incorporated into the revised manuscript where appropriate to strengthen the discussion and improve comparison with the literature. References that were not directly relevant to the objectives or methodological framework of the present study were not added. (Discussion-Paragraphs 2,3,5 and 6; References 20,23,24 and 27)

Reviewers' comments

Reviewer 1 Comment 1: In the introduction, although the main focus of the study is peri-implant bone healing, the rationale for including clinical periodontal parameters should be explained more clearly. In addition, if the statements regarding the value of fractal analysis in predicting implant success and the originality of the study are presented in a more cautious manner, the text will appear more balanced.

Response: Thank you for this valuable comment. We revised the Introduction to clarify the rationale for including clinical periodontal parameters and to present the role of fractal analysis in a more balanced and cautious manner. Specifically, we clarified that clinical periodontal parameters were included as secondary exploratory variables to provide additional clinical context for the local oral environment during early healing, whereas the primary focus of the study remained the radiographic assessment of peri-implant bone changes. We also softened the wording regarding the clinical value of fractal analysis and avoided overly strong statements concerning implant success prediction and the originality of the study. In the revised Introduction, the study aim is now presented more c

Attachment Submitted filename: Response to Reviewers.docx

# Peer review: Decision Letter 1

**DOI:** 10.1371/journal.pone.0352984.r003

**Authors:** Katibe Tugce Temur, Katibe Tugce Temur

20 May 2026

PONE-D-26-11186R1

Evaluation of early trabecular changes around implants using fractal analysis and panoramic indices

PLOS One

Dear Dr. keles,

Thank you for submitting your manuscript to PLOS ONE. After careful consideration, we feel that it has merit but does not fully meet PLOS ONE’s publication criteria as it currently stands. Therefore, we invite you to submit a revised version of the manuscript that addresses the points raised during the review process.

Please submit your revised manuscript by Jul 03 2026 11:59PM. If you will need more time than this to complete your revisions, please reply to this message or contact the journal office at plosone@plos.org. When you're ready to submit your revision, log on to https://www.editorialmanager.com/pone/ and select the 'Submissions Needing Revision' folder to locate your manuscript file.

Please include the following items when submitting your revised manuscript:

- A letter that responds to each point raised by the academic editor and reviewer(s). You should upload this letter as a separate file labeled 'Response to Reviewers'.
- A marked-up copy of your manuscript that highlights changes made to the original version. You should upload this as a separate file labeled 'Revised Manuscript with Track Changes'.
- An unmarked version of your revised paper without tracked changes. You should upload this as a separate file labeled 'Manuscript'.

If you would like to make changes to your financial disclosure, please include your updated statement in your cover letter. Guidelines for resubmitting your figure files are available below the reviewer comments at the end of this letter.

If applicable, we recommend that you deposit your laboratory protocols in protocols.io to enhance the reproducibility of your results. Protocols.io assigns your protocol its own identifier (DOI) so that it can be cited independently in the future. For instructions see: https://journals.plos.org/plosone/s/submission-guidelines#loc-laboratory-protocols. Additionally, PLOS ONE offers an option for publishing peer-reviewed Lab Protocol articles, which describe protocols hosted on protocols.io. Read more information on sharing protocols at https://plos.org/protocols?utm_medium=editorial-email&utm_source=authorletters&utm_campaign=protocols.

As the corresponding author, your ORCID iD is verified in the submission system and will appear in the published article. PLOS supports the use of ORCID, and we encourage all coauthors to register for an ORCID iD and use it as well. Please encourage your coauthors to verify their ORCID iD within the submission system before final acceptance, as unverified ORCID iDs will not appear in the published article. *Only*  the individual author can complete the verification step; PLOS staff *cannot*  verify ORCID iDs on behalf of authors.

We look forward to receiving your revised manuscript.

Kind regards,

Katibe Tugce Temur

Academic Editor

PLOS One

Journal Requirements:

If the reviewer comments include a recommendation to cite specific previously published works, please review and evaluate these publications to determine whether they are relevant and should be cited. There is no requirement to cite these works unless the editor has indicated otherwise.

Please review your reference list to ensure that it is complete and correct. If you have cited papers that have been retracted, please include the rationale for doing so in the manuscript text, or remove these references and replace them with relevant current references. Any changes to the reference list should be mentioned in the rebuttal letter that accompanies your revised manuscript. If you need to cite a retracted article, indicate the article’s retracted status in the References list and also include a citation and full reference for the retraction notice.

Additional Editor Comments:

Dear Authors,

Thank you for submitting your manuscript. The topic is interesting and clinically relevant; however, several issues should be addressed to improve the methodological clarity and scientific reliability of the study.

1. ROI selection and reproducibility

The description of ROI selection is not sufficiently clear or reproducible. Although the manuscript states that “the mesial and distal ROIs were positioned parallel to the implant surface along the peri-implant trabecular bone” and that the apical ROI was placed immediately apical to the implant apex, the exact anatomical and radiographic reference points used for ROI placement should be described in greater detail.

Due to the two-dimensional nature of panoramic radiographs, magnification, distortion, and superimposition are inevitable, particularly in images obtained at different follow-up intervals. These factors may especially affect fractal dimension measurements performed on small ROIs. Since the main objective of the study is to evaluate changes in bone structure, even minor differences in ROI positioning may influence the results. Therefore, the authors should provide a clearer and more reproducible explanation of ROI standardization.

In addition, the rationale for the selected ROI size and the specific methodological details derived from references 15 and 16 should be explicitly explained rather than only cited.

2. Terminology

Terminology should be used consistently throughout the manuscript. The terms “early structural changes,” “early healing,” and “trabecular remodelling” appear to be used interchangeably. The authors should select a single main conceptual framework and use it consistently throughout the title, abstract, introduction, discussion, and conclusion.

3. Language and punctuation

The language, grammar, and punctuation of the manuscript should be carefully reviewed. Several sentences would benefit from editing to improve clarity, academic flow, and readability.

4. Exclusion criterion related to crestal bone loss

The exclusion criterion “findings of crestal bone loss in follow-up images” should be reconsidered. Excluding cases with crestal bone loss may introduce selection bias and may shift the results in a more favorable direction. Since crestal bone loss is clinically relevant to peri-implant bone changes, the authors should either provide a clear justification for this exclusion criterion or consider including such cases with appropriate subgroup or sensitivity analysis.

5. Figure quality and resolution

The resolution and quality of the figures should be improved. The current figures are not sufficiently clear for evaluating ROI placement, measurement points, and radiographic details. Higher-resolution images should be provided, and all annotations, labels, and arrows should be made clearly visible. This is particularly important for figures demonstrating ROI selection and fractal analysis procedures.

[Note: HTML markup is below. Please do not edit.]

Reviewers' comments:

Reviewer's Responses to Questions

**Comments to the Author**

1. If the authors have adequately addressed your comments raised in a previous round of review and you feel that this manuscript is now acceptable for publication, you may indicate that here to bypass the “Comments to the Author” section, enter your conflict of interest statement in the “Confidential to Editor” section, and submit your "Accept" recommendation.

Reviewer #1: All comments have been addressed

Reviewer #2: (No Response)

**********

2. Is the manuscript technically sound, and do the data support the conclusions?

The manuscript must describe a technically sound piece of scientific research with data that supports the conclusions. Experiments must have been conducted rigorously, with appropriate controls, replication, and sample sizes. The conclusions must be drawn appropriately based on the data presented.

Reviewer #1: Yes

Reviewer #2: Partly

**********

3. Has the statistical analysis been performed appropriately and rigorously?

Reviewer #1: Yes

Reviewer #2: Yes

**********

4. Have the authors made all data underlying the findings in their manuscript fully available?

The PLOS Data policy requires authors to make all data underlying the findings described in their manuscript fully available without restriction, with rare exception (please refer to the Data Availability Statement in the manuscript PDF file). The data should be provided as part of the manuscript or its supporting information, or deposited to a public repository. For example, in addition to summary statistics, the data points behind means, medians and variance measures should be available. If there are restrictions on publicly sharing data—e.g. participant privacy or use of data from a third party—those must be specified.

Reviewer #1: Yes

Reviewer #2: No

**********

5. Is the manuscript presented in an intelligible fashion and written in standard English?

PLOS ONE does not copyedit accepted manuscripts, so the language in submitted articles must be clear, correct, and unambiguous. Any typographical or grammatical errors should be corrected at revision, so please note any specific errors here.

Reviewer #1: Yes

Reviewer #2: Yes

**********

6. Review Comments to the Author

Please use the space provided to explain your answers to the questions above. You may also include additional comments for the author, including concerns about dual publication, research ethics, or publication ethics. (Please upload your review as an attachment if it exceeds 20,000 characters)

Reviewer #1: (No Response)

Reviewer #2: 1. ‘The mesial and distal ROIs were positioned parallel to the implant surface along the peri-implant trabecular bone; the apical ROI was situated immediately apical to the implant apex. ROI placement was standardised using the implant contour, the implant apex and the adjacent root contours as reference points.’

The description of ROI selection is not sufficiently clear or reproducible. Due to the two-dimensional nature of panoramic radiographs, magnification, distortion and superimposition are inevitable in images taken at different time intervals. This situation may particularly affect FD measurements in small ROIs. The main focus of the study is to examine changes in bone structure. Minor changes in ROI selection may affect the results. Furthermore, there is no explanation regarding the points in references 15 and 16 cited in the methodology. Reference has been made to the size of the ROI.

2. Terminology must be consistent. The flow of the terms ‘early structural changes’, ‘early healing’ and ‘trabecular remodelling’ should be checked. A single main framework should be selected and used consistently.

3. The language and punctuation throughout the text should be reviewed again.

EXCLUSION CRITERIA

8. Findings of crestal bone loss in follow-up images.

Excluding this item may bias the study results in a positive direction.

**********

7. PLOS authors have the option to publish the peer review history of their article (what does this mean?). If published, this will include your full peer review and any attached files.

If you choose “no”, your identity will remain anonymous but your review may still be made public.

**Do you want your identity to be public for this peer review?**  For information about this choice, including consent withdrawal, please see our Privacy Policy.

Reviewer #1: No

Reviewer #2: No

**********

[NOTE: If reviewer comments were submitted as an attachment file, they will be attached to this email and accessible via the submission site. Please log into your account, locate the manuscript record, and check for the action link "View Attachments". If this link does not appear, there are no attachment files.]

To ensure your figures meet our technical requirements, please review our figure guidelines: https://journals.plos.org/plosone/s/figures

You may also use PLOS’s free figure tool, NAAS, to help you prepare publication quality figures: https://journals.plos.org/plosone/s/figures#loc-tools-for-figure-preparation.

NAAS will assess whether your figures meet our technical requirements by comparing each figure against our figure specifications.

# Author comment: Author response to Decision Letter 2

**DOI:** 10.1371/journal.pone.0352984.r004

26 May 2026

Dear Academic Editor and Reviewers,

We thank you for the careful evaluation of our manuscript entitled “Evaluation of early trabecular changes around implants using fractal analysis and panoramic indices.” We appreciate the constructive comments and have revised the manuscript accordingly. The major revisions include a more detailed and reproducible ROI standardization protocol, harmonization of terminology throughout the manuscript, clarification of the crestal bone loss-related exclusion criterion, improvement of figure quality and annotations, and provision of anonymized measurement data as S1 Data. Point-by-point responses are provided below.

Response to Academic Editor and Reviewer #2

Comment 1: ROI selection and reproducibility

The description of ROI selection is not sufficiently clear or reproducible. Although the manuscript states that “the mesial and distal ROIs were positioned parallel to the implant surface along the peri-implant trabecular bone” and that the apical ROI was placed immediately apical to the implant apex, the exact anatomical and radiographic reference points used for ROI placement should be described in greater detail.

Due to the two-dimensional nature of panoramic radiographs, magnification, distortion, and superimposition are inevitable, particularly in images obtained at different follow-up intervals. These factors may especially affect fractal dimension measurements performed on small ROIs. Since the main objective of the study is to evaluate changes in bone structure, even minor differences in ROI positioning may influence the results. Therefore, the authors should provide a clearer and more reproducible explanation of ROI standardization.

In addition, the rationale for the selected ROI size and the specific methodological details derived from references 15 and 16 should be explicitly explained rather than only cited.

Response: We thank the editor/reviewer for this important comment. We agree that ROI selection is a critical methodological step in fractal analysis performed on panoramic radiographs, because magnification, geometric distortion, and anatomical superimposition may influence FD measurements, particularly when small peri-implant ROIs are used. In response, we substantially revised the Fractal analysis procedure subsection of the Methods to provide a clearer and more reproducible description of ROI placement, standardization, baseline–follow-up matching, and ROI-size rationale.

Specifically, we clarified that baseline and 3-month panoramic radiographs were evaluated side by side to reproduce ROI placement at comparable peri-implant levels. Because baseline radiographs were obtained before implant placement, we further clarified that the implant long axis and the perpendicular apical reference line passing through the implant apex were first defined on the 3-month radiograph using the visible implant contour and apex. The corresponding baseline ROI locations were then reproduced on the preoperative radiograph by matching the implant recipient site with stable anatomical and radiographic landmarks, including adjacent root contours, alveolar crest morphology, edentulous ridge configuration, surrounding trabecular pattern, and region-specific anatomical boundaries such as the maxillary sinus floor, mandibular canal, or mental foramen when present. We also clarified that side-by-side assessment was used only to identify corresponding anatomical locations for ROI placement and not to visually judge changes in FD values.

We also added a geometric standardization protocol based on the implant long axis and the perpendicular apical reference line. ROI-1, ROI-2, and ROI-3 were defined as the mesial, distal, and apical peri-implant trabecular ROIs, respectively. ROI-1 and ROI-2 were oriented parallel to the implant long axis, with their inferior borders aligned with the apical reference line, and were positioned in the closest available mesial and distal peri-implant trabecular bone adjacent to the implant surface without including the implant threads or implant surface. ROI-3 was positioned immediately apical to the implant apex, with its superior border aligned with the same apical reference line and centered on the implant long axis.

We further clarified the exclusion boundaries during ROI placement. The implant surface, implant threads, cortical bone, lamina dura, adjacent roots, sinus floor, mandibular canal, radiopaque restorative materials, and areas of anatomical superimposition or image artifact were excluded from the ROIs. We also added that when the predefined ROI could not be reproducibly placed within trabecular bone without including these structures, that region was not measured. This rule was applied to both baseline and follow-up images to reduce measurement variability related to panoramic distortion and superimposition.

The rationale for the selected ROI dimensions was also expanded. The 25 × 50 pixel mesial and distal ROIs were selected to sample the narrow peri-implant trabecular zone along the implant surface while minimizing inclusion of the implant margin, adjacent root structures, or cortical borders. The 50 × 50 pixel apical ROI was selected to sample the trabecular area immediately apical to the implant apex, where a broader trabecular compartment was usually available. These ROI dimensions were kept fixed across patients and time points to obtain comparable measurements and to provide sufficient trabecular information for box-counting analysis. We also clarified that this approach was selected with reference to previous dental fractal analysis studies using fixed ROI dimensions and to evidence indicating that ROI size, configuration, and placement may influence FD measurements.

In addition, Fig 1 and its legend were revised to more clearly illustrate and define the standardized ROI placement. The revised Fig 1B now provides an enlarged visual representation of the peri-implant ROI placement protocol, including the implant long axis and the perpendicular apical reference line passing through the implant apex. The figure legend was also updated to explain that the corresponding baseline ROI locations were reproduced by side-by-side matching of stable anatomical landmarks, and to identify the location of ROI-1, ROI-2, and ROI-3 in relation to these reference lines.

Finally, we added a statement to the limitations section acknowledging that, despite the use of fixed ROI dimensions and reproducible radiographic landmarks, minor variations in ROI positioning cannot be completely excluded because of the inherent magnification, geometric distortion, and anatomical superimposition associated with panoramic radiography. This limitation is now explicitly stated to guide cautious interpretation of subtle FD changes. (Materials and methods: Fractal analysis procedure; Figure Legends: Figure 1; Discussion: Paragraph 7)

Comment 2: Terminology should be used consistently throughout the manuscript. The terms “early structural changes,” “early healing,” and “trabecular remodelling” appear to be used interchangeably. The authors should select a single main conceptual framework and use it consistently throughout the title, abstract, introduction, discussion, and conclusion.

Response: We thank the editor/reviewer for this important comment. We agree that the terminology should be used consistently throughout the manuscript and that the terms “early structural changes,” “early healing,” and “trabecular remodelling” should not be used interchangeably.

We retained the title because it already uses the neutral term “early trabecular changes,” which is consistent with the radiographic nature of the study and does not imply direct evidence of osseointegration or histological remodeling. However, in response to the comment, we revised the abstract, introduction, results, discussion, and conclusion to harmonize the terminology throughout the manuscript.

The main conceptual framework of the revised manuscript is now early trabecular changes, more specifically early peri-implant trabecular structural changes where appropriate. The term “early healing” is now used only to describe the clinical time interval, namely the 3-month unloaded healing period, rather than as the primary radiographic outcome. Similarly, terms such as “trabecular remodelling” were replaced where appropriate with more neutral terms such as “trabecular structural changes” or “radiographic trabecular structural changes.”

We also revised interpretive statements to avoid overinterpretation of the fractal analysis findings. In particular, we clarified that fractal analysis detects radiographic changes in trabecular microarchitecture and should not be interpreted as direct evidence of osseointegration or histological remodeling. These revisions were made to improve conceptual consistency and to ensure that the terminology accurately reflects the radiographic and exploratory nature of the study findings. (Abstract; Introduction: Paragraphs 4 and 5; Results: Paragraph 2; Discussion: Paragraphs 1-5 and 6-7; Conclusions)

Comment 3: Language and punctuation. The language, grammar, and punctuation of the manuscript should be carefully reviewed. Several sentences would benefit from editing to improve clarity, academic flow, and readability.

Response: We thank the editor/reviewer for this comment. We have carefully revised the manuscript for language, grammar, punctuation, clarity, and academic flow. Long or potentially ambiguous sentences were edited, and several sections were rephrased to improve readability, particularly the Abstract, Methods, Discussion, and Conclusion.

As part of this revision, we also harmonized terminology throughout the manuscript and revised interpretive statements to avoid overstatement of the radiographic findings. In addition, minor punctuation, spacing, abbreviation, and formatting errors were corrected throughout the text.

We believe that these revisions have improved the overall clarity, consistency, and readability of the manuscript.

Comment 4: Exclusion criterion related to crestal bone loss. The exclusion criterion “findings of crestal bone loss in follow-up images” should be reconsidered. Excluding cases with crestal bone loss may introduce selection bias and may shift the results in a more favorable direction. Since crestal bone loss is clinically relevant to peri-implant bone changes, the authors should either provide a clear justification for this exclusion criterion or consider including such cases with appropriate subgroup or sensitivity analysis.

Response: We thank the editor/reviewer for this important comment. We agree that excluding cases solely because crestal bone loss is present on follow-up radiographs could introduce selection bias and may shift the results in a more favorable direction. In response, we revised this exclusion criterion to clarify that crestal bone loss was not used as an exclusion criterion by itself.

In the revised manuscript, this exclusion criterion was reworded to indicate that radiographs were excluded only when crestal bone loss, peri-implant radiolucency, cortical disruption, or related radiographic changes prevented reproducible placement of the predefined trabecular ROIs. We also clarified that cases were not excluded solely on the basis of clinically detectable crestal bone loss; exclusion was applied only when radiographic changes prevented standardized ROI placement without including cortical defects, radiolucent areas, the implant surface, or anatomical superimposition.

This clarification was made to distinguish a technical radiographic exclusion criterion related to ROI reproducibility from a clinical exclusion criterion based on peri-implant bone status. Because the primary outcome of the study was based on FD measurements obtained from predefined trabecular ROIs, reliable and reproducible ROI placement was necessary to reduce measurement variability related to panoramic distortion, anatomical superimposition, and altered peri-implant anatomy.

In addition, we added a statement to the limitations section acknowledging that, although this approach may have reduced measurement variability, it may also limit the generalizability of the findings to implants with radiographically stable peri-implant bone conditions. (Materials and methods: Paragraphs 5 and 6; Discussion: Paragraph 7)

Comment 5: Figure quality and resolution. The resolution and quality of the figures should be improved. The current figures are not sufficiently clear for evaluating ROI placement, measurement points, and radiographic details. Higher-resolution images should be provided, and all annotations, labels, and arrows should be made clearly visible. This is particularly important for figures demonstrating ROI selection and fractal analysis procedures.

Response: We thank the editor/reviewer for this comment. We agree that high-quality figures are essential for evaluating ROI placement, measurement points, radiographic landmarks, and the fractal analysis workflow. In response, we revised the figure files and improved the visibility of annotations, labels, arrows, ROI boundaries, reference lines, and measurement lines.

Fig 1 was revised into two panels to improve readability at publication size. Fig 1A now shows the panoramic morphometric measurements, including MCW, ML, PMI, and the mental foramen reference. Fig 1B provides an enlarged view of the peri-implant ROI placement. In this enlarged panel, the ROI boundaries, reference lines, arrows, and labels were made more distinct to improve visualization of ROI-1, ROI-2, and ROI-3. The figure legend was revised accordingly to define the blue longitudinal line as the implant long axis and the blue transverse line as the perpendicular apical reference line, and to clarify the standardized positions of the ROIs.

Fig 2 was also reviewed and prepared in higher resolution to improve the visibility of the fractal analysis workflow, panel labels, processing steps, and box-counting output. Revised high-resolution TIFF files have been uploaded with the revised manuscript. (Figures 1 and 2; Figure Legends)

Reviewer #1 Comment: All comments have been addressed

Response: We thank Reviewer #1 for the positive assessment and for indicating that all previous comments have been addressed.

Additional comment. Data availability

Reviewer comment: Has the author made all data underlying the findings in their manuscript fully available?

Reviewer #1: Yes

Reviewer #2: No

Response: We thank the reviewer for this comment. We acknowledge the importance of making all data underlying the findings available in accordance with the PLOS ONE data policy. In the revised submission, we have provided the anonymized measurement dataset underlying the findings as S1 Data.

The S1 Data file includes the de-identified clinical and radiographic measurement data used in the analyses, including FD values, panoramic morphometric measurements, periodontal parameters, and relevant grouping variables. The dataset therefore provides the data points underlying the reported means, standard deviations, and statistical comparisons.

To protect participant confidentiality, all potentially identifying information, including patient names, institutional record numbers, radiographic identifiers, exact imaging dates, and other direct or indirect identifiers, has been removed from the dataset. Raw panoramic radiographs are not publicly available because they contain potentially identifying patient information and were obtained from retrospective clinical archives under ethics committee approval.

Accordingly, the Data Availability Statement was revised to clarify that all anonymized measurement data underlying the findings are provided as Supporting Information, whereas raw radiographic images are not publicly available due to participant privacy considerations. (Data availability; S1 Data)

Attachment Submitted filename: Response_to_Reviewers_auresp_2.docx

# Peer review: Decision Letter 2

**DOI:** 10.1371/journal.pone.0352984.r005

**Authors:** Katibe Tugce Temur, Katibe Tugce Temur, Katibe Tugce Temur

18 Jun 2026

Evaluation of early trabecular changes around implants using fractal analysis and panoramic indices

PONE-D-26-11186R2

Dear Authors,

Thank you for submitting the revised version of your manuscript.

The manuscript has been carefully re-evaluated together with the reviewers’ comments and your responses to the previous concerns. The revised version has improved considerably, particularly in terms of methodological clarity, interpretation of the findings, and discussion of the study limitations.

The remaining concerns have been adequately addressed, and the conclusions are now presented in a sufficiently cautious manner. In particular, fractal analysis is appropriately interpreted as a supplementary radiographic method for detecting early trabecular changes, rather than as direct evidence of osseointegration.

Therefore, I consider the manuscript scientifically suitable for publication, pending completion of the outstanding technical requirements.

Congratulations to the authors.

Sincerely,

# Editor report: Acceptance letter

**DOI:** 10.1371/journal.pone.0352984.r006

**Authors:** Katibe Tugce Temur, Katibe Tugce Temur, Katibe Tugce Temur

PONE-D-26-11186R2

PLOS One

Dear Dr. keles,

I'm pleased to inform you that your manuscript has been deemed suitable for publication in PLOS One. Congratulations! Your manuscript is now being handed over to our production team.

At this stage, our production department will prepare your paper for publication. This includes ensuring the following:

* All references, tables, and figures are properly cited

* All relevant supporting information is included in the manuscript submission,

* There are no issues that prevent the paper from being properly typeset

You will receive further instructions from the production team, including instructions on how to review your proof when it is ready. Please keep in mind that we are working through a large volume of accepted articles, so please give us a few days to review your paper and let you know the next and final steps.

Lastly, if your institution or institutions have a press office, please let them know about your upcoming paper now to help maximize its impact. If they'll be preparing press materials, please inform our press team within the next 48 hours. Your manuscript will remain under strict press embargo until 2 pm Eastern Time on the date of publication. For more information, please contact onepress@plos.org.

You will receive an invoice from PLOS for your publication fee after your manuscript has reached the completed accept phase. If you receive an email requesting payment before acceptance or for any other service, this may be a phishing scheme. Learn how to identify phishing emails and protect your accounts at https://explore.plos.org/phishing.

If we can help with anything else, please email us at customercare@plos.org.

Thank you for submitting your work to PLOS ONE and supporting open access.

Kind regards,

PLOS ONE Editorial Office Staff

on behalf of

Dr. Katibe Tugce Temur

Academic Editor

PLOS One
